# Molecular Mechanisms in Metal Oxide Nanoparticle–Tryptophan Interactions

**DOI:** 10.1021/acs.inorgchem.3c03674

**Published:** 2024-04-29

**Authors:** Alexandra Nefedova, Fredric G. Svensson, Alexander S. Vanetsev, Peter Agback, Tatiana Agback, Suresh Gohil, Lars Kloo, Tanel Tätte, Angela Ivask, Gulaim A. Seisenbaeva, Vadim G. Kessler

**Affiliations:** † Institute of Physics, University of Tartu, W.Ostwaldi 1, 50411 Tartu, Estonia; ‡ Department of Solid State Physics, Ångström Laboratory, Uppsala University, Box 35, SE-75103 Uppsala, Sweden; § Department of Molecular Science, BioCenter, Swedish University of Agricultural Sciences, Box 7015, 75007 Uppsala, Sweden; ∥ Applied Physical Chemistry, KTH Royal Institute of Technology, Teknikringen 30, SE-100 44 Stockholm, Sweden; ⊥ Institute of Molecular and Cell Biology, University of Tartu, Riia 23, 51010 Tartu, Estonia

## Abstract

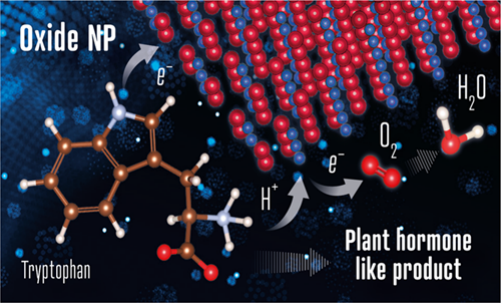

One of the crucial metabolic processes for both plant and animal kingdoms is the oxidation of the amino acid tryptophan (TRP) that regulates plant growth and controls hunger and sleeping patterns in animals. Here, we report revolutionary insights into how this process can be crucially affected by interactions with metal oxide nanoparticles (NPs), creating a toolbox for a plethora of important biomedical and agricultural applications. Molecular mechanisms in TRP–NP interactions were revealed by NMR and optical spectroscopy for ceria and titania and by X-ray single-crystal study and a computational study of model TRP–polyoxometalate complexes, which permitted the visualization of the oxidation mechanism at an atomic level. Nanozyme activity, involving concerted proton and electron transfer to the NP surface for oxides with a high oxidative potential, like CeO_2_ or WO_3_, converted TRP in the first step into a tricyclic organic acid belonging to the family of natural plant hormones, auxins. TiO_2_, a much poorer oxidant, was strongly binding TRP without concurrent oxidation in the dark but oxidized it nonspecifically via the release of reactive oxygen species (ROS) in daylight.

## Abstract 2

The molecular mechanism of tryptophan oxidation in the interaction with metal oxide nanoparticles has been revealed through NMR, X-ray single-crystal, and theoretical investigations.

## Introduction

Tryptophan (TRP) is an essential amino acid with key metabolic functions in both the animal and plant kingdoms. In plants, it is involved, via its oxidation, in the formation of plant growth hormones, auxins,^1^ while in animals it plays a crucial role in energy storage and transfer processes, being a precursor of nicotinamide adenine dinucleotide (NAD^+^) in the kynurenine pathway,^2,3^ influencing the immune system and brain function.^3^ It is involved in mammals in the regulation of sleep^4^ and hunger.^5^

Recent studies have indicated that the interaction of TRP with oxide nanoparticles (NPs) may have played an important role in the origin of life, shaping the formation and function of the first living cells.^6^ NPs were demonstrated to affect the metabolic pathways of TRP,^7^ in particular, via the regulation of the generation and activity of reactive oxygen species (ROS).^8−10^ The effects of NPs were mostly addressed for TiO_2_ and CeO_2_ in plant metabolism, where the accelerated growth of plants treated with these NPs was attributed to TRP oxidation leading to auxin generation.^11−13^

The effects of ceria as a reduction catalyst and an antioxidant were also sought in several studies to be related to its effect on TRP metabolism in animals. In particular, ceria was proposed as a new therapeutic tool in the treatment of liver diseases.^14^ Ceria NPs were found to inhibit the differentiation of neural stem cells.^15^ CeO_2_ and iron oxide TRP composites were proposed for application as food and feed additives,^16^ contrast agents in cell investigations,^17^ and cosmetics.^18^ Special attention has been paid to tungsten oxide TRP composites that have exhibited antibacterial and anticancer activity.^19^ Some attention has even been paid to surface complexes of TRP and some other amino acids on metal oxides as chromophores.^20^

In view of the strong interest to the effects of the TRP interaction with NPs, surprisingly little effort has been made on the investigation of its molecular mechanisms. Except some optical spectroscopy studies^21^ and the work of covalently bound tryptophan,^22^ we were, to the best of our knowledge, not able to find any structural and mechanistic characterization of TRP–NP interactions. In the present study, we report the NMR characterization of the TRP interaction with bare and capped ceria and titania surfaces and the bare tungsten oxide surface (in the form of a POM model, phosphotungstic acid) and an X-ray single-crystal study and theoretical investigation of the latter complex with TRP, which provided direct evidence for a new mechanism of the direct (not via intermediate reactive oxygen species, ROS) oxidation of the amino acid via simultaneous electron and proton transfer.

## Experimental Section

All chemicals were purchased from Sigma-Aldrich and used without further purification.

Titanium oxide colloids surface-stabilized by either triethanolamine or by lactate anions were obtained as reported previously in ref (23). The sample of TiO_2_–I was produced by the modification of titanium ethoxide with dry triethanolamine (mixing 5.0 mL of liquid Ti(OEt)_4_ and 1.5 mL of liquid N(C_2_H_4_OH)_3_ until the formation of a clear yellowish solution) with subsequent dropwise addition on vigorous stirring of a hydrolyzing solution produced by mixing 0.5 M HNO_3_ (0.5 mL) with EtOH (2.0 mL). This procedure resulted in the NP starting solution with a TiO_2_ concentration of 120 mg/g as established by TGA. The other applied sample, TiO_2_–II, was produced by dilution with Milli-Q water of the solution of the commercially available TiBALDH chemical, a 50 wt % solution with respect to (NH_4_)_8_Ti_4_O_4_(OCOCHOCH_3_)_8_·4H_2_O. The initial concentration of TiO_2_ was estimated according to the established solution equilibrium as 45 mg/mL.^24^ TEM and DLS characterizations of TiO_2_–I and TiO_2_–II have been reported earlier in refs (23) and (24), respectively.

Cerium dioxide stable colloidal samples with positive (nano-CeO_2_(+)) and negative (nano-CeO_2_(−)) surface charges were produced using techniques previously reported in ref (25). In short, for the synthesis of nano-CeO_2_(+) nanoparticles, diammonium cerium(IV) nitrate was hydrolyzed in the presence of hexamethylenetetramine (HMTA) at 180 °C for 30 min in the microwave-hydrothermal device (Berghof Speedwave 4, 2.45 GHz, 1000 W). The product was washed with deionized water and redispersed by ultrasonication. As a result, nanoparticles with an almost “bare” surface carrying a positive charge were obtained. For the synthesis of nano-CeO_2_(−) nanoparticles, cerium(III) nitrate was hydrolyzed at room temperature in the presence of ammonia with simultaneous oxidation by oxygen from the air. After synthesis, the colloidal solution was centrifuged, washed, and redispersed by ultrasonication. This method allowed the production of ceria nanoparticles stabilized by citrate ions and therefore carrying a large negative charge. TEM and DLS characterizations of nano-CeO_2_(+) and nano-CeO_2_(−) have been reported earlier in ref (25).

### Synthesis of the POM Model

Ca. 0.200 g of phosphotungstic acid (ca. 0.11 mmol) was dissolved in 2 mL of distilled water and then a solution of 3 equiv of TRP (0.065 g) in 2 mL of 1 M HCl was added. The obtained dark red solution was left for crystallization overnight, providing 0.25 g (ca. 94% yield) of reddish black crystals of the desired product, (HTRP)_3_PW_12_O_40_·5H_2_O(s), and a practically colorless supernatant.

### Photo-Oxidation Study

In contrast to the solution of ceria NPs and the mother liquor over the POM complex, the solutions of titania NPs with TRP kept in the dark did not contain any oxidation products of TRP according to the NMR data. To evaluate the effect of potential photocatalytic processes with titania, the samples of TiO_2_–I, TiO_2_–II, and nonsurface-capped TiO_2_ produced by the rapid hydrothermal synthesis^26^ (10 mg with respect to dry TiO_2_) were put into a Petri dish with 2 mL of 20 mM solution of TRP. The mixtures were set for illumination by simulated daylight for 72 h at room temperature. The dried samples were extracted with 0.5 mL of DMSO–D6 and investigated by ^1^H NMR.

### Characterization

#### NMR Study

The NMR experiments were acquired on a Bruker Advance III spectrometer operating at 14.1 T, equipped with a cryo-enhanced QCI-P probe at a temperature of 298 K. For assignment of the chemical shifts of the oxidized TRP product, Bruker standard pulse sequences of 2D TOCSY, HSQC, HMBC, and NOESY were used. Spectra were processed with TopSpin 4.2.0.

All spectra where TRP was titrated with NPs were acquired in 5 mm tubes (a final volume of 0.500 mL) with the internal ^1^H chemical shift standard, 0.1 mM DSS (4,4-dimethyl-4-silapentane-1-sulfonic acid), and ^13^C chemical shifts were referenced indirectly to the ^1^H standard using a conversion factor derived from the ratio of NMR frequencies. Additionally, the synthetic ERETIC (electronic reference to access in vivo concentrations) signal at −0.40 ppm was implemented in every 1D ^1^H proton spectrum. Its intensity was calibrated to the concentration of 0.3 mM. The ERETIC signal was used as the external standard for intensity calibration in titration experiments.

DOSY experiments of the free TRP and (HTRP)_3_PW_12_O_40_·5H_2_O(s) complex in DMSO–D6 solution were performed with the Bruker standard pulse sequence using bipolar gradient pulses for diffusion, 2 spoil gradients, and a longitudinal eddy (LED) delay.^27^ The relaxation delay time, D1, was 3 s, and the diffusion time (Δ) was 50–300 ms according to the properties of samples. The duration of the pulse field gradient (δ/2) was adjusted to be in the range of 800–2000 μs in order to obtain 2–5% residual signal with the maximum gradient strength. The delay for gradient recovery was 0.2 ms, and the eddy current delay was 5 ms. The gradient strength was incremented in 32 steps from 2 to 95% of its maximum value in a linear ramp. The diffusion coefficient was calculated using Bruker Dynamic center 2.8.3 software.

#### Sample Preparations for the Titration of Tryptophan with NPs for NMR Study

Stock solutions of TiO_2_–I (2 mM), TiO_2_–II (2 mM), L-TRP (10 mM), and DSS (25 mM) in H_2_O were prepared. From the TiO_2_–I and TiO_2_–II solutions, 1, 2, 3, 4, 8, 16, 32, 64, and 128 μL were pipetted into separate 1.5 mL Eppendorf vials and freeze-dried overnight. To each vial, 2 μL of DSS solution, 25 μL of TRP solution, and 473 μL of D_2_O were added. The samples were then put into an ultrasonic bath for 10 min at 25 °C and thereafter transferred into 5 mm 4 in. NMR tubes.

For CeO_2_(+) and CeO_2_(−), the stock solutions were 90 μM. Samples were made by pipetting 2.8, 5.6, 11.1, 26.8, and 38.9 μL into vials and then adding 25 μL of the tryptophan stock solutions and 2 μL of the DSS stock solutions and enough D_2_O to make each sample 500 μL. The samples were then transferred into 5 mm 4 in. NMR tubes. The pH was checked to be 6.0 for all CeO_2_ and TiO_2_ samples in H_2_O/D_2_O. Crystals of POM were dissolved in 500 μL of DMSO and transferred into a 5 mm 4 in. NMR tube. No pH control was thus possible for the POM solution, but it was supposedly rather acidic in view of the presence of HTRP^+^ cations.

LC-MS/MS analyses were performed by using a high-resolution Q Exactive HF Orbitrap mass spectrometer system (Thermo Fisher Scientific, Inc.). An Agilent 1290 Infinity II liquid chromatography system (Agilent Technologies, Santa Clara, CA) was coupled to the mass spectrometer. The separation of the metabolites was performed on a Luna Omega 100 mm × 4.6 mm 3 μm PS C18 100 Å LC column (Phenomenex, Værløse, Denmark) maintained at 25 °C. The mobile phases consisted of (A) water and (B) acetonitrile, both containing 0.2% formic acid. An isocratic run with the mobile phase (B) at 2% and a flow rate of 0.4 mL/min was used. The following generic MS tune parameters were used: spray voltage, 3.5 kV; capillary temperature, 350 °C; sheath gas flow, 45 arbitrary units; auxiliary gas flow, 15 arbitrary units; and probe heater temperature, 320 °C. The automatic gain control (AGC) target value and maximum injection time used for the full MS scan were 3e6 and 200 ms, respectively. The Q Exactive′s instrumental method of full MS/AIF was used to collect MS and MS/MS data, which were then analyzed using Thermo′s FreeStyle analysis program. The MS data detected the presence of kynurenine and two diastereoisomers of dioxindolylalanine and 3-hydroxypyrroloindole carboxylic acid, as depicted in Figure S5 of the Supporting Information.

#### UV–Vis Spectroscopy

The spectra in the regions 200–450 and 300–1000 nm were measured at different concentrations with a Thermo Scientific Multiskan SkyHigh microplate spectrophotometer, using an ethanol–water 1:1 mixture as the reference.

#### FTIR Spectroscopy

The spectra were recorded for KBr tablets in transmission mode and for powders of TRP adsorbed on NPs in ATR mode using a PerkinElmer Spectrum 100 instrument.

#### DLS

The determination of hydrodynamic particle size and surface charge of the particles (ζ-potential) was carried out with a Malvern Panalytical Zetasizer Ultra instrument.

#### X-ray Crystallography

A suitable crystal was selected in Nujol oil under a microscope and placed on an amorphous cellulose needle onto a goniometer head. The data collection was carried out with a Bruker D8 Quest ECO instrument in the 2-theta range 4–58° using the Apex-IV program package. Data integration was made in the range 4–50.05° (1.0 Å resolution). Full details of data collection, solution, and refinement are provided in Supporting Table 1. C_33_H_41_N_6_O_51_PW_12_, orthorhombic, space group *P*2_1_2_1_2_1_, *a* = 14.970(4), *b* = 16.670(4), *c* = 26.833(6) Å, *V* = 6696(2) Å, *Z* = 4 at *T* = 295(2) K. The structure was solved by direct methods with all W and P atoms appearing in the initial solution. All other non-hydrogen atoms were found in subsequent difference Fourier syntheses. All non-hydrogen atoms were refined first in anisotropic and then in anisotropic approximation. Hydrogen atoms were introduced by geometrical calculation and included into the final refinement in isotropic approximation with temperature factors fixed at 1.200 times that for the carbon or nitrogen atoms they were attached to. Final discrepancy factors were *R*1 = 0.0294, w*R*2 = 0.0714 for 10841 observed [Fo > 4sigma(Fo)] and *R*1 = 0.0351, w*R*2 = 0.0745 for all 11810 data. Brief details of the experiment are available in Tables TS1 and TS2 (Supporting Information). Full details of data collection and refinement are available free of charge from the Cambridge Crystallographic Data Centre (CCDC) at http://www.ccdc.cam.ac.uk citing the reference numbers 2277146 for the compound reported here and 2277147 for the Mo analogue as supplementary contribution.

### Computational Details

**Figure 1:**
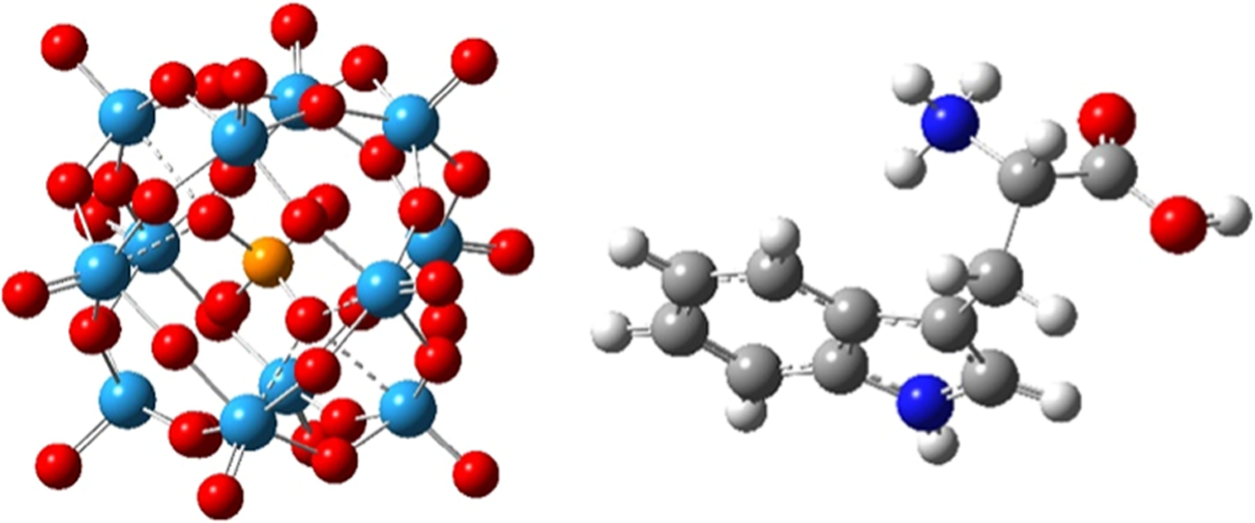
Molecular structures of the polyoxometalate ion (W_12_PO_40_^3–^, left) and the protonated TRP amino acid (HTRP^+^, right) studied here.

All calculations were performed using the program package Gaussian16 (Rev. C.01).^28^ Visualizations were performed using GaussView 6,^29^ and natural bond order (NBO) estimates were performed with NBO7 as interfaced with Gaussian16.^30^ Density functional theory (DFT) computations were performed using the hybrid density functional cam-B3LYP including long-range corrections, and the basis set for W was based on the Stuttgart–Dresden–Cologne MDF60 effective-core potential (ECP) and a valence space of double-ζ quality.^31^ The lighter elements were modeled using basis sets of 6-311G quality amended by diffuse and polarization functions. The initial structures of W_12_PO_40_^3–^ and protonated TRP (HTRP^+^) were taken from the orthorhombic (*P*2_1_2_1_2_1_) crystal structure of (HTRP)_3_W_12_PO_40_·5H_2_O. The structures were geometrically optimized before analysis. Implicit solvent effects for water as the solvent were included using the polarizable continuum model (PCM)^32^ as implemented in Gaussian16 (Figure 1).

## Results and Discussion

The aim of the present study was to gain insight into how different kinds of metal oxide nanoparticles actually interacted with TRP and to reveal whether there were formed inner or outer-sphere complexes and whether the oxidation of TRP could proceed via its specific transformation, the nanozyme effect, or whether the major track would be a nonspecific oxidation by reactive oxygen species (ROS) generated by NPs. We have selected two modifications of two representative nanomaterials, broadly discussed in connection with surface complexation and redox activity, ceria and titania. For each material, we have chosen one modification that essentially had an unprotected surface (CeO_2_(+) and TiO_2_–I) and another with the surface capped with strongly attached chelating carboxylate ligands (citrate for CeO_2_(−) and lactate for TiO_2_–II) to evaluate the effect of surface capping on the amino acid interaction. Both titania and ceria particles applied in this work are ca. 3.5 nm in size and have been reported and characterized earlier in detail (see refs (23,24) for titania and ref (25) for ceria). As potential molecular reference models, we have selected Keggin POM species, [PM_12_O_40_]^3–^, M = Mo, W, the smallest available spherical metal oxide nanoparticles with a size of 1.04 nm.

The applied strategy was first to screen the NMR spectra for different NP:TRP ratios in order to detect potential complexation equilibria and determine if chemical transformations of TRP did take place. The aim was then to identify if such a transformation was specific in its nature and how it could occur on a molecular level, exploiting insights into the structure of the molecular model compound.

The NMR study of the TRP interactions with TiO_2_–I or TiO_2_–II NPs was carried out at pH 6.0.

The detailed characterization of TiO_2_ colloids is described elsewhere.^23^ The nanoparticles possess crystalline cores covered by a relatively thin amorphous shell with a mean size of about 3.5 nm. The zeta-potentials for these two types of NPs were −11 and −23 mV, respectively. Both types of particles form completely transparent clear colloid solutions that can be diluted with Milli-Q water without any measurable changes in their characteristics. 1D proton, ^1^H, spectra in D_2_O solution at pH 6.0 of the TRP ligand (0.5 mM) in a mixture with different ratios of TiO_2_–I and TiO_2_–II NPs showing aromatic protons in regions between 7.8 and 7.0 ppm and an expended region between 7.77 and 7.68 ppm for TRP H6 are presented in Figures S2(A,B) and 2(A,B), respectively. The normalized intensities, *I*/*I*^o^, of the H6 proton resonance of TRP at 7.728 ppm vs the ratio (*R*) of TRP:TiO_2_–I and TRP:TiO_2_–II are presented in Figure S3.

**Figure 2:**
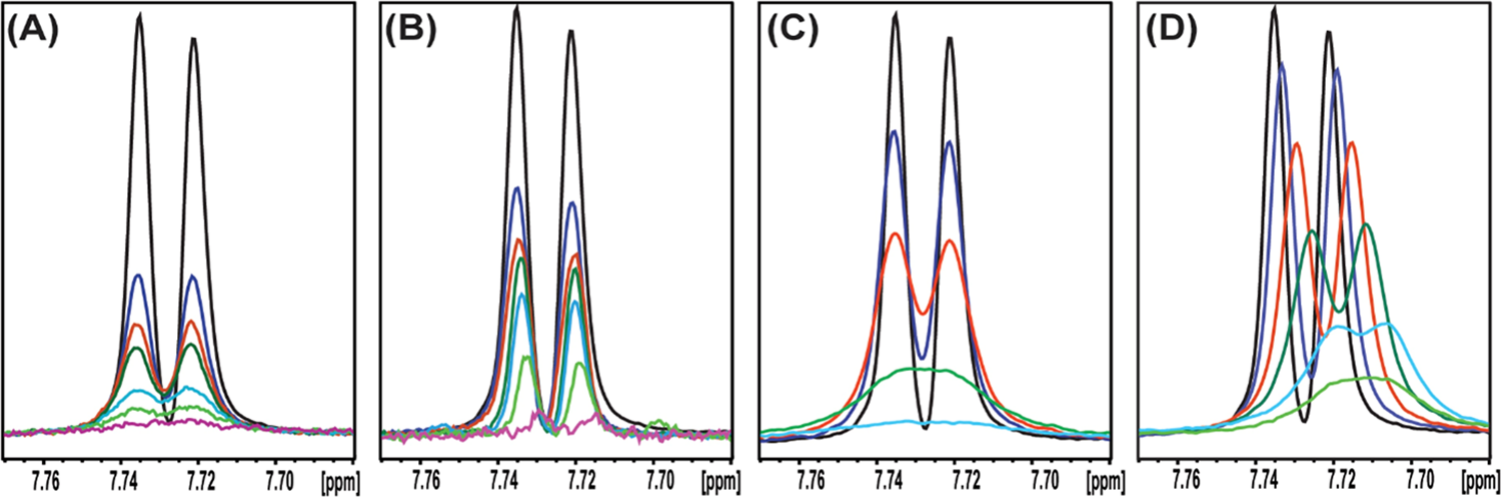
Titration of 0.5 mM TRP with TiO_2_–I (A), TiO_2_–II (B), CeO_2_(−)(C), and CeO_2_(+)(D) showing the H6 proton resonance of TRP at 7.728 ppm. The following colors of curves obtained at different ratios of NP/TRP were used. (A, B) TRP:TiO_2_–I or TiO_2_–II:0.5 mM:0 mM (black); 0.5 mM:0.031 mM (blue); 0.5 mM:0.0625 mM (red); 0.5 mM:0.125 mM (green); 0.5 mM:0.250 mM (light blue); 0.5 mM:0.5 mM (light green); and 0.5 mM:1.0 mM (rose). (C, D) TRP:CeO_2_(−)(C)/or CeO_2_(+)(D):0.5 mM:0 mM (black); 0.5 mM:0.0005 mM (blue); 0.5 mM:0.001 mM (red); 0.5 mM:0.002 mM (green); 0.5 mM:0.005 mM (light blue); and 0.5 mM:0.007 mM (light green).

It is evident from Figures 2(A,B) and S1(A,B) that the line width of resonances of TRP under titration with either TiO_2_–I or TiO_2_–II hardly changed. For a two-state model,^26^ the decrease in the signal intensity without line broadening indicated a strong binding of TRP to NPs and that chemical exchange between free TRP and the bound complex TRP:NP occurred under a slow exchange condition. In this condition, resonances belonging to the complex of TRP:NP cannot be observed due to the line broadening from the slow tumbling motion of the complex. The observed signals belong to unbound free TRP left in solution. Using an ERETIC signal, *I*^o^, as an intensity reference, the dependence of normalized intensities, *I*/*I*^o^, from *R* in the range 0 and 2 shows linearity, as presented in Figure S2, indicating a zeroth-order adsorption reaction. Nevertheless, it is puzzling that free TRP is still detected at a ratio of 1 and 2, indicating that a more complex process is going on during the titration and that a simple two-state model could not be applied. At this level of study, we can only hypothesize that the concentration of NPs under the influence of TRP could be reduced due to their reassembly into larger NPs. In corroboration with this hypothesis is the fact that in the fresh stock solution of TiO_2_–I and TiO_2_–II, the size of NPs was estimated by DLS as ca. 3.5 nm. However, on addition of TRP and especially with time, the fractions of larger NPs became observed. The major size shifted to ca. 8 nm and much larger fractions about 150–200 nm appeared for TiO_2_–I, and about 150–200 and ca. 800 nm (the latter even dominating after 3 days of storage) for TiO_2_–II (see Figure S3). Additionally, it is remarkable that the slopes of the curve *I*/*I*^o^ vs *R* for both TiO_2_–I and TiO_2_–II NPs are very similar even though they obtained using different capping ligands. This allowed us to conclude that TRP was in competition interaction with the ligand on TiO_2_–II NPs, forming strongly bound inner-sphere complexes on the surface for both titania NP materials.

The NMR study of the tryptophan interaction with nano-CeO_2_(−) and nano-CeO_2_(+) was carried out at pH 6.0.

The detailed characterization of CeO_2_ colloids is described elsewhere.^25^ The nanoparticles are crystalline, with a mean size slightly below 3.5 nm. The surface charge (zeta-potential) of nanoparticles in the colloidal solution for nano-CeO_2_(+) is +41 ± 2 mV and for nano-CeO_2_(−) is −53 ± 4 mV, ensuring the high stability of water colloids.

**Figure 3:**
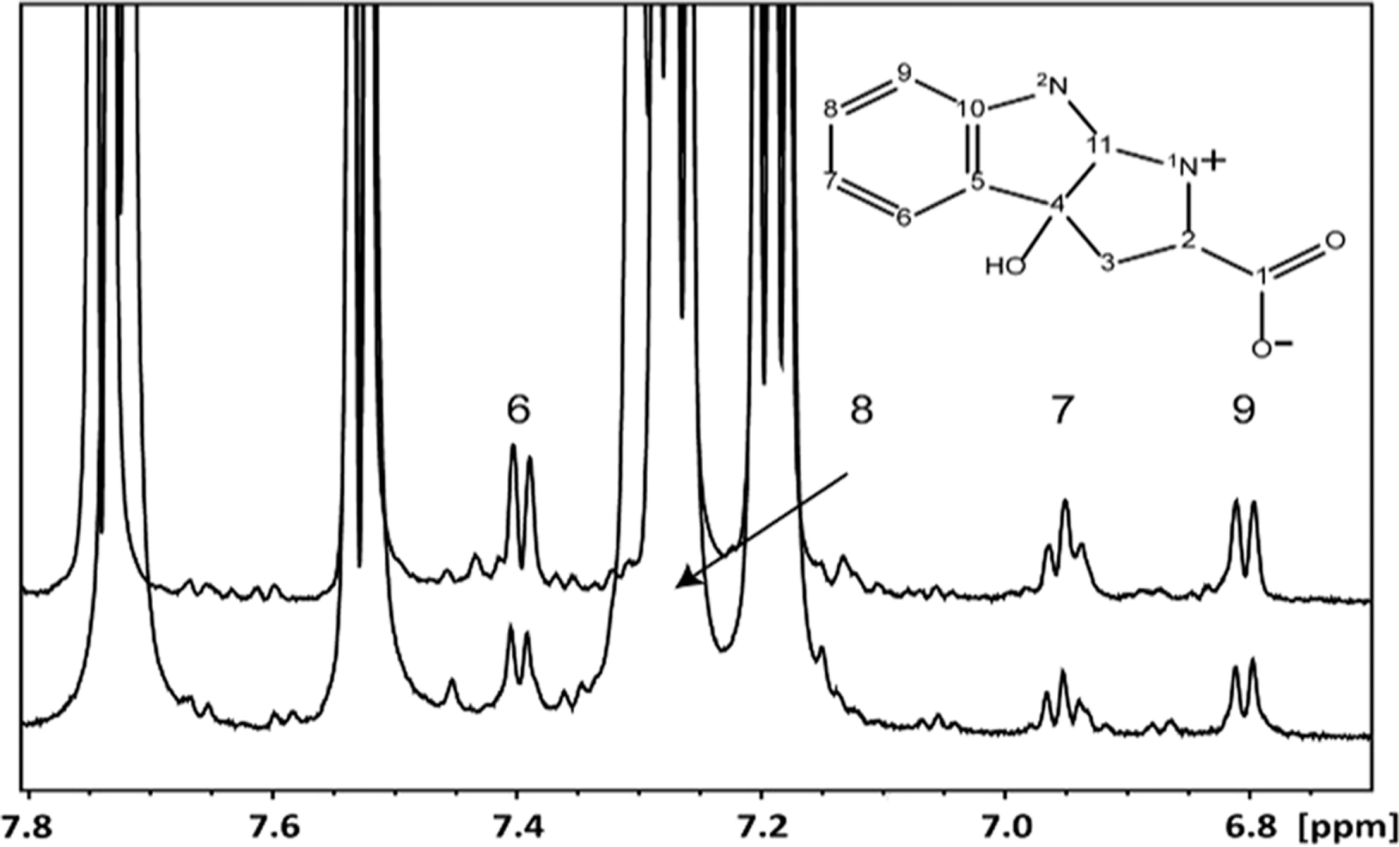
1D ^1^H spectrum with assignment of the proton resonances of the oxidized product of TRP obtained in a mixture with CeO_2_(−)(C) NP. Expanded spectra in the aromatic 8.0–6.5 ppm region are presented. The assignment, performed using the superposition of the HMBC and HSQC spectra (Figure S4), and numbering are shown according to the structure on the panel. The lower normalized spectrum corresponds to overnight treatment of 1 mg/mL TRP in the presence of 8 mg/mL CeO_2_(−) immediately after centrifugation and gives 2.9 ± 0.5% of the primary product by integration. The upper spectrum is for the sample after removal of ca. 90% CeO_2_(−) by centrifugation and 1 month storage at 5 °C. The primary oxidized product content is 6.4 ± 0.4% by integration.

1D proton, ^1^H, spectra in D_2_O solution at pH 6.0 of the TRP ligand (0.5 mM) in a mixture with different concentrations of nano-CeO_2_(−) and nano-CeO_2_(+) are presented in Figure S1(C,D) and the expanded region between 7.77 and 7.68 ppm for TRP H6 is presented in Figure 2(C,D). The main difference between the TRP resonances upon addition of nano-CeO_2_(−) and nano-CeO_2_(+) compared to TiO_2_–I and TiO_2_–II is that the NMR spectra of the former exhibit a decrease in the signal intensity and an increase in line broadening already at a very low concentration of NPs at the ratio *R* = 0.001, as shown in Figure 2(C,D) for the H6 proton. Additionally, the titration by nano-CeO_2_(+) shows upfield chemical shifts of the aromatic protons (Figure 2(D)). Based on this data, we propose that TRP is involved in fast exchange^33^ with nano-CeO_2_(−) and nano-CeO_2_(+) NPs through the creation of weakly bound complexes, i.e., outer-sphere ones. Remarkably, some new sets of resonances have been observed in the NMR spectra of the mixture of TRP with CeO_2_(−)(C) NPs as illustrated on the 1D ^1^H spectrum shown in Figure 3A,B. The same distinct individual product was observed even in the mother liquor of the POM–TRP complex. It is known that TRP can be metabolized into various forms under different conditions. However, predicting the specific type of TRP modification under certain conditions is not straightforward. This is why we conducted an identification study of the single uniquely created product that we detected. We have assigned this new product which accumulates with time to be a distinct oxidized form of TRP. Assignment of the resonances of the new product has been performed based on a set of 2D spectra including COSY, HSQC, NOESY, and HMBC, as presented in Figure S4A,B. Additional proof of the proposed structure of oxidized TRP presented in Figure 3 was obtained by LC-MC experiments.

**Scheme 1:**
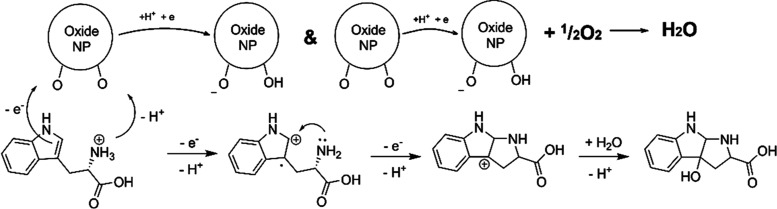
Proposed Molecular Mechanism of TRP Oxidation Based on Observed Structural Features of the POM–TRP Complex, Theoretical Calculation, and the Identified Nature of the Reaction Product The latter is apparently generated in a neutral form, but will then, of course, transform into a zwitterion.

The HPLC-MS (see Figure S5) investigation of the sample contributed to the identification of a very distinct major reaction product with a characteristic retention time of 7.1 min on the applied column and the molecular mass of 221.0949 Da, corresponding to a mono-oxygenated form of TRP. The first fragmentation product of this compound is formed via the loss of a water molecule. The structure of the first oxidation product deduced from the NMR data features an additional heterocycle formed via cyclization of the α-amino group of the amino acid with the indole unit of the TRP molecule that has thus lost its aromaticity via the release of a proton and an electron simultaneously in two steps (see Scheme 1). The double bond transforms then to a single one via the addition of a water molecule with a hydroxyl group ending with being attached to the carbon atom C4 from the original indole ring (Scheme 1). The observed specific oxidation occurring in the dark indicates the so-called nanozyme activity analogous for CeO_2_ and POM in this case. It is important to mention that while the oxidation is chemically specific leading to 3-hydroxypyrroloindol carboxylic acid (PIC),^7^ it is not stereospecific with 2 stereoisomers of the primary product distinguishable in LC-MS. Even the second product, dioxindolylalanine, appearing after about 1 month of storage in the NMR spectrum, is composed of 2 stereoisomers resolved in LC-MS. The third product in the order of its accumulation in the sample is kynurenine, containing only one isomer inherited from the original l-tryptophan structure. It is interesting to mention that these three products have earlier been observed in comparable amounts in 3 different pathways in the oxidation of TRP by the photogenerated singlet oxygen. The main reaction product here, PIC,^7^ appears in this particular case as a single primary oxidation product (see the Supporting Information, Figure S9). It bears clear resemblance in its structure and potential chemical reactivity to natural auxins, the plant growth-enhancing hormones,^34^ and has been associated with strong bioactivity,^35^ making CeO_2_ and other potentially oxidative oxide NPs attractive candidates for the in situ nanozyme production of auxins from TRP. It is interesting to note that the formation of PIC as a single primary oxidation product of TRP, via the reaction mechanism resembling that presented by this work, was actually observed in rather small quantities on the photochemical oxidation of TRP in the polarized magnetic field^36,37^ or simply by the singlet molecular oxygen generated photochemically in the presence of specific dyes.^38^ In the reaction of TRP with oxidative NPs in this work, the PIC species are generated under ambient conditions, without special physical excitation and in darkness. The second oxidation product appears after storage of the centrifuged reaction mixture for over 1 month at 5 °C in darkness. Its structure, elucidated by HMBC NMR (see the Supporting Information, Figure S10), features the breakdown of the third heterocycle in PIC. The complete degradation of TRP into a complex mixture of products was observed after 1 year of storage.

While the photochemical synthesis of PIC under relatively harsh acidic conditions has been optimized and reported earlier in literature,^39^ the results reported in this work with its slow generation under ambient conditions in darkness can be of special interest, in particular, for agricultural applications.

It is important to note that no change in the spectrum of TRP was observed in the presence of either TiO_2_–I or TiO_2_–II NPs even after 72 h of continuous irradiation by simulated solar light. Both materials turned bluish, but no additional signals could be observed in the aromatic domain of the ^1^H NMR signals. The effect was most probably due to the presence of antioxidant ligands, triethanolamine and lactate, in the case of TiO_2_–I and TiO_2_–II NPs, respectively. To prove this hypothesis, we have carried out a parallel experiment with bare TiO_2_ anatase nanoparticles of comparable size (below 10 nm) produced by rapid hydrothermal synthesis.^26^ In this latter case, a multitude of signals with comparable intensity was observed in the aromatic region of the ^1^H NMR spectrum (see Figure S8), indicating a nonspecific random oxidation process caused, apparently, by photochemically generated ROS.

### (HTRP)_3_PM_12_O_40_·5H_2_O, M = Mo, W Complex

The interaction of Keggin phosphotungstate with tryptophan apparently involves the charge transfer phenomenon in view of the deep wine red coloration of the solution after mixing colorless solutions of the reagents and the almost black color of the solid reaction product (HTRP)_3_PW_12_O_40_·5H_2_O forming small and often intergrown plate-like crystals. In the case of the molybdenum derivative, the solution remains colorless and the precipitating complex is dark bluish black colored. The single crystals for (HTRP)_3_PMo_12_O_40_·5H_2_O in the form of bluish black flattened needles could only be obtained in a counter-diffusion experiment, where the solutions of TRP and H_3_Mo_12_O_40_ hydrate were frozen in layers on top of each other and left for slow thawing over 48 h in a Dewar vessel.

The produced model tungsten compound is practically insoluble in water, but sparingly soluble in an ethanol:water = 1:1 mixed solvent, producing a dark, slightly brownish red color. In its UV spectrum, one can see a distinct phosphotungstate signature with three well-defined strongly absorbing bands at 208, 222, and 268 nm with a shoulder at 289 nm. All bands are shifted approximately 50 nm to a shorter wavelength region compared to recently reported pure phosphotungstic acid and its complexes with bipyridine isomers.^40^ This shift was supposedly due to the charge transfer effects in the POM–amino acid interactions. The visible region is characterized by a broad band in the region of 400–740 nm with the intensity decreasing with the increase in the wavelength. It features shoulders at 480 and 640 nm (see Figure S6).

### NMR Study of the *(*HTRP)_3_PW_12_O_40_·5H_2_O Complex in DMSO–D6 Solution

**Figure 4:**
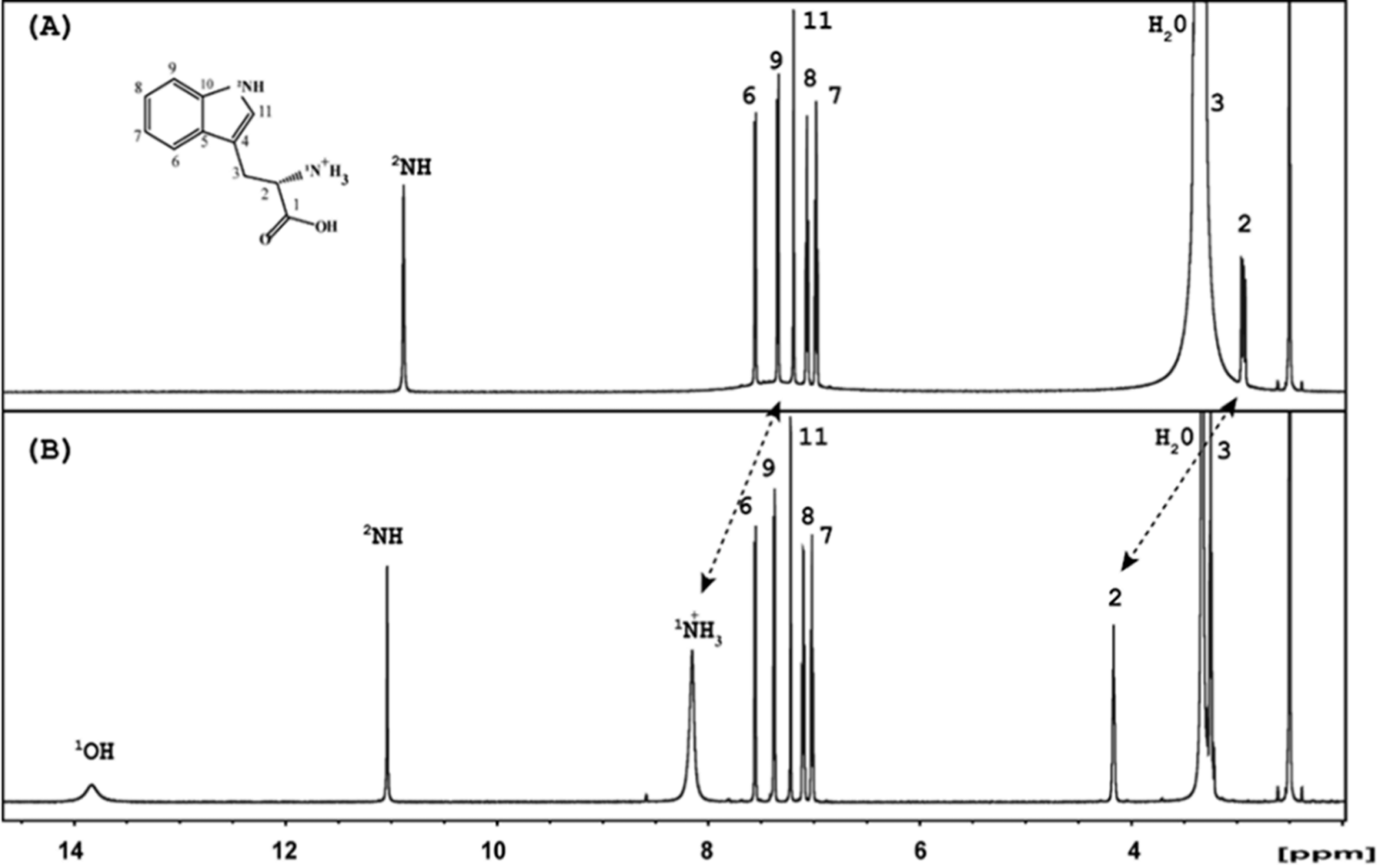
1D ^1^H spectra of free TRP (A) and its complex (HTRP)_3_PW_12_O_40_·5H_2_O (B). Assignments of the TRP in the complex performed based on HSQC and HMBC experiments (Figure S7) are shown on top of the proton resonances.

Due to the poor solubility of the (HTRP)_3_PW_12_O_40_·5H_2_O complex in water, the NMR study of this complex was performed in DMSO–D6. As shown in Figure 4(A,B), 1D proton, ^1^H, spectra of free TRP and its complex (HTRP)_3_PW_12_O_40_·5H_2_O in DMSO–D6 are presented, respectively. For assignment of the ^1^H and ^13^C resonances, 2D HSQC, HMBC spectra (Figure S7(A)), and the COSY spectrum were used. In the ^1^H spectrum of the complex (HTRP)_3_PW_12_O_40_·5H_2_O vs free HTRP, large downfield chemical shifts (CSs) are detected for protons of the backbone, H2, and the amino group, ^1^NH_2_, at 1.22 and 0.82 ppm, respectively (Figure 4(A,B)). This allows us to suggest that these protons are involved in the interaction with PW_12_O_40_. Additionally the relaxation properties of the protons of the amino ^1^NH_2_ and ^1^OH groups are significantly changed. Indeed, in free TRP, strong line broadening is observed for the protons of the amino group ^1^NH_2_ but not if TRP is involved in the complex with PW_12_O_40_. This corroborates our proposal that ^1^NH_2_ protons are involved in hydrogen bonding with PW_12_O_40_ in a DMSO solution. Noteworthily, the ^1^NH_2_ signals are observed in the ^1^H spectrum (Figure 4B) as a superposition of three lines with different intensities, indicating that the ^1^NH_2_ group is protonated forming an ammonium ion in TRP involved in complexation. Additionally, the observation in the (HTRP)_3_PW_12_O_40_·5H_2_O complex of the acid proton resonance of the ^1^COOH group at 13.85 ppm (Figure 4B) indicates that the ^1^COOH group is involved in strong hydrogen bonding with the NPs. Neither large changes in the CS nor line broadening are observed for protons belonging to the aromatic part of TRP. Keeping this in mind, we performed additional experiments to prove that three TRP molecules are coordinated as HTRP^+^ cations to [PW_12_O_40_]^3–^ anions in solution, as has been detected in the crystalline form.

Diffusion NMR can determine the variation of the diffusion coefficient or the hydrodynamic size of a target molecule before and after the formation of NP complexes. The self-diffusion coefficients of free TRP and the (HTRP)_3_PW_12_O_40_·5H_2_O complex in DMSO–D6 were obtained from ^1^H DOSY NMR. The diffusion coefficient of pure TRP is (7.68 ± 0.06) × 10^–10^ m^2^/s. Upon addition of the (HTRP)_3_PW_12_O_40_· 5H_2_O complex, the diffusion coefficient decreased significantly to (1.74 ± 0.01) × 10^–10^m^2^/s. The lower (HTRP)_3_PW_12_O_40_·5H_2_O complex diffusion coefficients indicate that in solution, there is the free-state TRP and HTRP^+^-[PW_12_O_40_]^3–^ ion pair complex that coexist in solution in exchange.^41^ Unfortunately, the specific complex diffusion coefficient could not be determined due to its very low concentration in solution.

### Molecular Structure of the Model Compound

**Figure 5:**
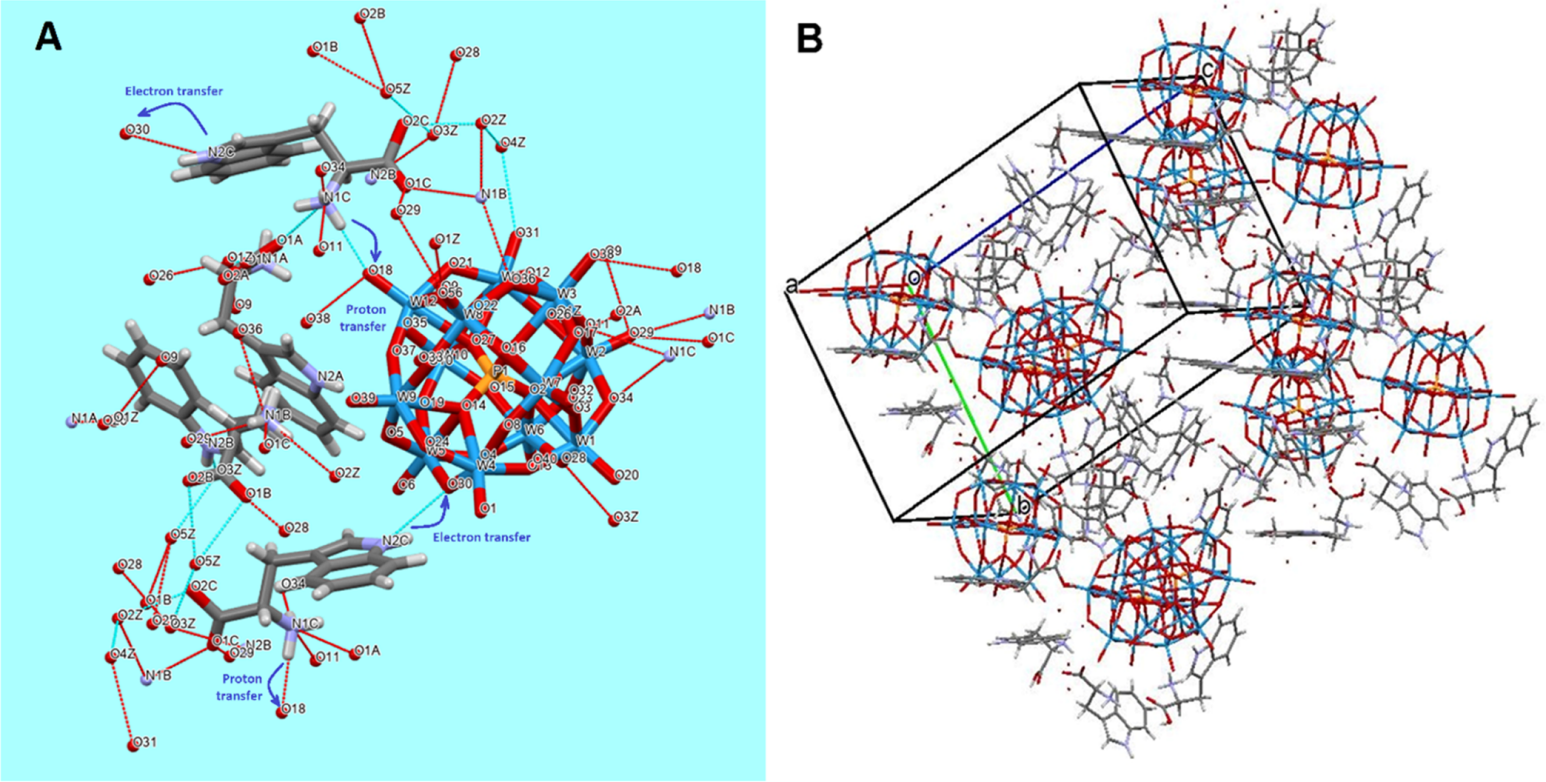
Molecular structure of (HTRP)_3_PW_12_O_40_·5H_2_O with hydrogen bonds indicated (A) and its packing motif (B).

The structure of (HTRP)_3_PW_12_O_40_·5H_2_O (Figure 5A) involves a structurally undisturbed α-Keggin [PW_12_O_40_]^3–^ anion, surrounded on one side by 3 protonated tryptophan cations (see the Supporting Information Figure S11), hydrogen-bonded to a water molecule denoted as O(1A), and, on the other side, by four water molecules O2A–O5A. The latter water molecules are hydrogen-bonded to the carboxylic groups of the amino acid species (O1C–O5A 2.759(8) Å), while the ammonium cations of the TRP molecules are connected via somewhat longer hydrogen bonds (2.985(3)–2.044(8) Å) to the oxygen atoms on the surface of the POM species, together with some shorter ones, to interstitial water molecules (N1D-O1A 2.720(8) Å). An important question about the mechanism of the charge transfer may, hypothetically, be answered by the observed relatively short hydrogen bond between the nitrogen atom in the indole aromatic core of the terminal amino acid group N2B (NH) and the bridging oxygen atom O9 of the POM (N2B–O9 2.990(8) Å). This link stays apparently for the first electron transfer event (see Scheme 1), while the proton transfer should be effectuated from the ammonium −NH_3_^+^ group that is also strongly hydrogen-bonded to the POM.

The packing in the isomorphous structures of the (HTRP)_3_PM_12_O_40_·5H_2_O, M = Mo, W, complexes (Figure 5B) features a combination of both dense packing motifs for the POM units as spherical particles and of π–π stacking of the indole units of TRP, representing a distinct contrast to known oligo-glycine POM complexes, where the pseudo hexagonal dense packing of the POM units was the only factor defining the crystal structure construction.^42,43^

### Theoretical Evaluation of Reaction Pathways for the Model Compound

The model structures for W_12_PO_40_^3–^ and HTRP^+^ and their oxidized/reduced analogues were all geometrically optimized without symmetry constraints (the formal point group *C*_1_). The W_12_PO_40_^3–^ structure is quite close to T symmetry, but optimization within that point group offers very little difference in terms of final structure end energies as compared to the one optimized in *C*_1_. All geometrically optimized structures represent at least the local minima on the expected complex potential energy surface, as shown by a vibrational frequency analysis displaying no negative eigenvalues of the Hessian. An NBO charge analysis shows that any of the 12 terminal oxygen atoms of W_12_PO_40_^3-/4-^ represent feasible acceptors upon protonation; they all have similar negative charges. In this context, it can be noted that the oxygen atoms with the highest negative charges in the polyoxometalate systems are the four atoms directly coordinating to the central phosphorus(V) atom.

Relevant energies for a general survey of potential spontaneous reactions are shown below. Using the computed total energies, thermodynamically corresponding to internal energies, reactions 1–3 can be formulated as shown below:
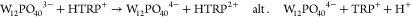
1Δ*E* = +14.76 eV, alt. +21.19 eV.
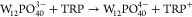
2Δ*E* = +11.12 eV.
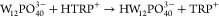
3Δ*E* = +2.46 eV, or +2.68 eV in the water solvent.

It is clear that all of the reactions 1–3 are energetically unfavorable. Since pressure–volume work is expected to be small, the Δ*E* energies obtained computationally are expected to be reasonably good approximations of the corresponding changes in enthalpy. It is also unlikely that large changes in entropy would convert any of the reactions from being unfavorable to becoming thermodynamically favorable. However, it is notable that reaction 3, representing a quite common proton-coupled electron transfer (PCET) in biological systems, makes the electron transfer almost feasible; +2.46 eV corresponds to +237 kJ/mol. The observed short hydrogen bond N2B–O9 may be an indication of how proton-coupled electron transfer actually may take place.

Taking on a frontier-orbital view, the energies of the highest occupied and lowest unoccupied molecular orbitals (HOMO–LUMO energies) of the reactants all indicate that the spontaneous transfer of an electron from any variant of TRP to the POM, corresponding to an oxidation of the TRP entity, is unlikely (see Table TS3).

The W_12_PO_40_^3–^ ion was also exposed to an analysis of expected predominant transfers from the singlet ground state to singlet and triplet excited states by using time-dependent DFT (TD-DFT). A complex, multiatom system, such as W_12_PO_40_^3–^, will have several molecular orbitals similar in energy and character, and close to both its formal HOMO and LUMO. Therefore, one should not expect a clean electron excitation from the HOMO to the LUMO to represent the predominant lowest energy transfer. Indeed, the results from TD-DFT analysis show several singlet-to-singlet and singlet-to-triplet transfers of similar energy involving contributions from several orbitals close to the frontier orbitals. The lowest energy transfer is about 4.1 eV, corresponding to an excitation wavelength of about 300 nm, in the UV region of light. The POM is thus expected to have a slight yellow color. Theoretical computations for multinegative species, such as the POM anions in this study, tend to give positive, nonphysical LUMO energies and too high HOMO energies. Thus, direct comparison of orbital energies to identify a charge transfer pathway becomes less reliable. However, implicit solvent effects can be introduced using a dielectric field of the solvent (water) also including different protonation states of the POM; see Table S3. A comparison of frontier-orbital energies, for instance, for W_12_PO_40_^3–^(aq) and HTRP^+^(aq) in reaction 3, at least makes a light-induced oxidation of HTRP^+^ by the POM highly feasible.

As for the TRP ions and molecule, the nonoxidized forms of HTRP^+^ and TRP show the lowest energy transfer at about 390 nm in the UV region, both being singlet-to-triplet transitions. The oxidized ions HTRP^2+^ and TRP^+^ exhibit more complex light-absorption characteristics where the resulting states cannot be assigned to specific spin states. Both oxidized species show weak features in the red/near-infrared region, but the most intense transition is in the range 530–560 nm. This means that the oxidation of TRP should be expected to be associated with a change from weak yellow to a deeper color, most likely blue.

### Binding Modes of the TRP Ligand According to FTIR Data

An important aspect in getting insight into the actual mechanism of TRP oxidation by a nanozyme process in contact with NPs is the actual state of the TRP substrate. In the investigated molecular model, showing an apparent charge transfer, the tryptophan ligands were present in the protonated form as HTRP^+^ cations in the solid state.

**Figure 6:**
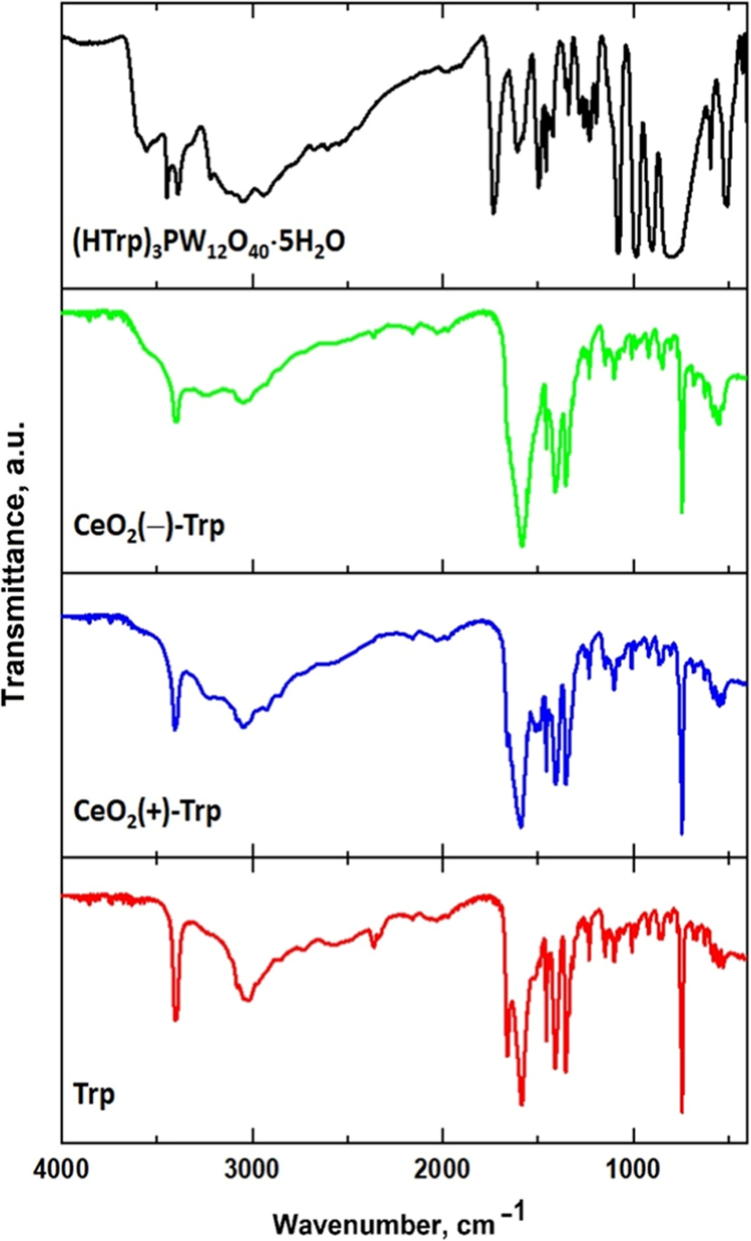
FTIR spectra of the solids isolated by drying buffered TRP solutions and that of the pure POM–TRP complex.

The pH of the solution for (HTRP)_3_PW_12_O_40_·5H_2_O could not be detected reliably because of its poor solubility, but for earlier investigated derivatives of oligo-glycines, it was about 3.0.^42,43^ On the contrary, the selective oxidation of TRP by ceria NPs was observed at pH = 6.0–7.0, a value exceeding the isoelectric point of TRP, which is 5.89, implying that the actual species in solution were the zwitterionic form with a fraction of the anionic one, featuring a deprotonated amino group. The analysis of the FTIR spectra of buffered TRP samples isolated by drying from solutions with and without added ceria nanoparticles (see Figure 6) shows a distinct contrast to that of (HTRP)_3_PW_12_O_40_·5H_2_O in both the bands corresponding to vibrations of the carboxylate group and those for stretching N–H vibrations. While the spectrum of the POM complex displays, as expected for the HTRP^+^ cations, a high-energy band for the C–O stretching mode at 1735 cm^–1^ along with two lower energy bands at 1589 and 1609 cm^–1^, corresponding to fully protonated C(=O)OH groups (apparently involved in hydrogen bonding), those of buffered TRP and its complexes with ceria show partially resolved double peaks at 1586 and 1676 cm^–1^ for TRP, 1592 and 1660 cm^–1^ for CeO_2_(+)-TRP, and 1585 and 1660 cm^–1^.

In the N–H stretching region, in the spectra of buffered samples, one can observe only one strong peak at 3403–3406 cm^–1^, corresponding to the indole fragment, while in that of the POM complex, two strong lines are present, at 3392 and at 3447 cm^–1^,^44^ indicating that the indole units there have different characteristics dependent on whether they are involved in the hydrogen bond with a charge transfer or not. The N–H stretching frequencies^45^ for the ammonium ions also differ considerably, being in the range 3243–3253 cm^–1^ for buffered TRP and CeO_2_–TRP samples and 3337 cm^–1^ for the POM–TRP complex. The observed differences in the FTIR spectra indicate that the TRP ligands in the outer-sphere complexes of ceria are connected to the surface via hydrogen bonding to both the carboxylate anion and to the −NH group of the indole unit. The latter is then expected to represent the channel for electron and proton transfer behind the nanozyme action of ceria (as indicated in Scheme 1). This mechanism is expected to be facilitated by electrostatic reasons for the zwitterionic and especially anionic forms of TRP, strengthening the insights obtained from the evaluation of the POM–TRP molecular model.

It is important to mention that we have obtained, in this case, the first direct structural evidence for the ways of TRP amino acid binding to a NP surface and also the first direct observation of how a specific nanozyme-type oxidation pathway occurs for TRP. Earlier studies^46,47^ were only based on indirect spectroscopic data and theoretical calculations lacking reliable structural models. A preprint summarizing preliminary results of the study described here has been deposited at Research Square.^48^

## Conclusions

Metal oxide NPs, dependent on their redox properties, were found to either act as nanozymes, specifically oxidizing TRP into an auxin-like heterocyclic organic acid, or, for weaker oxidants acting as photocatalysts, randomly oxidize TRP into a variety of different products by the action of photogenerated ROS. The key principle in the nanozyme action appears to be the formation of an outer-sphere NP–TRP complex based on hydrogen bonding involving the NH fragment of the TRP indole ring. The direct insight into this mechanism was obtained by combination of (HTRP)_3_PM_12_O_40_·5H_2_O, M = Mo, W, molecular model structure determination, and theoretical evaluation. The chemistry of these species clearly indicates a charge transfer mechanism. The latter is then facilitated for redox-active oxide NPs due to the deprotonation of the TRP ligand under pH-neutral conditions.

## Supporting Information Available

NMR spectra for the titration of materials with TRP, normalized signal intensities for NMR spectra in titration experiments, DLS data for the particle size distribution in materials with added TRP, 2D NMR spectra for the identification of the oxidation product, mass spectra of the oxidation product, UV–vis spectra of the model POM compound, the 2D NMR spectrum of the model POM compound in DMSO, NMR spectra of the photo-oxidation products, and tables with the results of X-ray single-crystal and theoretical investigations (PDF)

## Abbreviations

TRP: tryptophan
