# Supplementary material for: Molecular Mechanisms in Metal Oxide Nanoparticle–Tryptophan Interactions
Source: Inorg Chem. 2024 Apr 29;63(19):8556–66. doi: 10.1021/acs.inorgchem.3c03674 (PMC11094791; doi:10.1021/acs.inorgchem.3c03674)
Supplement: Supplementary file 1 — ic3c03674_si_001.pdf [file ic3c03674_si_001.pdf]

# Supplementary Materials

## Molecular mechanisms in metal oxide nanoparticle Tryptophan interactions

Alexandra Nefedova,[I] Fredric G. Svensson,[II] Alexander S. Vanetsev,[I]* Peter Agback,[ǂ] Tatiana Agback,[ǂ] Suresh Gohil,[ǂ] Lars Kloo,[illegible] Tanel Tätte,[I] Angela Ivask,[˧] Gulaim A. Seisenbaeva,[ǂ] Vadim G. Kessler[ǂ]*

[I] Institute of Physics, University of Tartu, W.Ostwaldi 1, 50411 Tartu, Estonia. alexander.vanetsev@ut.ee; [II] Department of Solid State Physics, Ångström Laboratory, Uppsala University, Box 35, SE-75103 Uppsala, Sweden; [ǂ] Department of Molecular Science, BioCenter, Swedish University of Agricultural Sciences, Box 7015, 75007 Uppsala, Sweden. vadim.kessler@slu.se; [illegible] Applied Physical Chemistry, KTH Royal Institute of Technology, Teknikringen 30, SE-100 44 Stockholm, Sweden; [˧] Institute of Molecular and Cell Biology, University of Tartu, Riia 23, 51010 Tartu, Estonia.

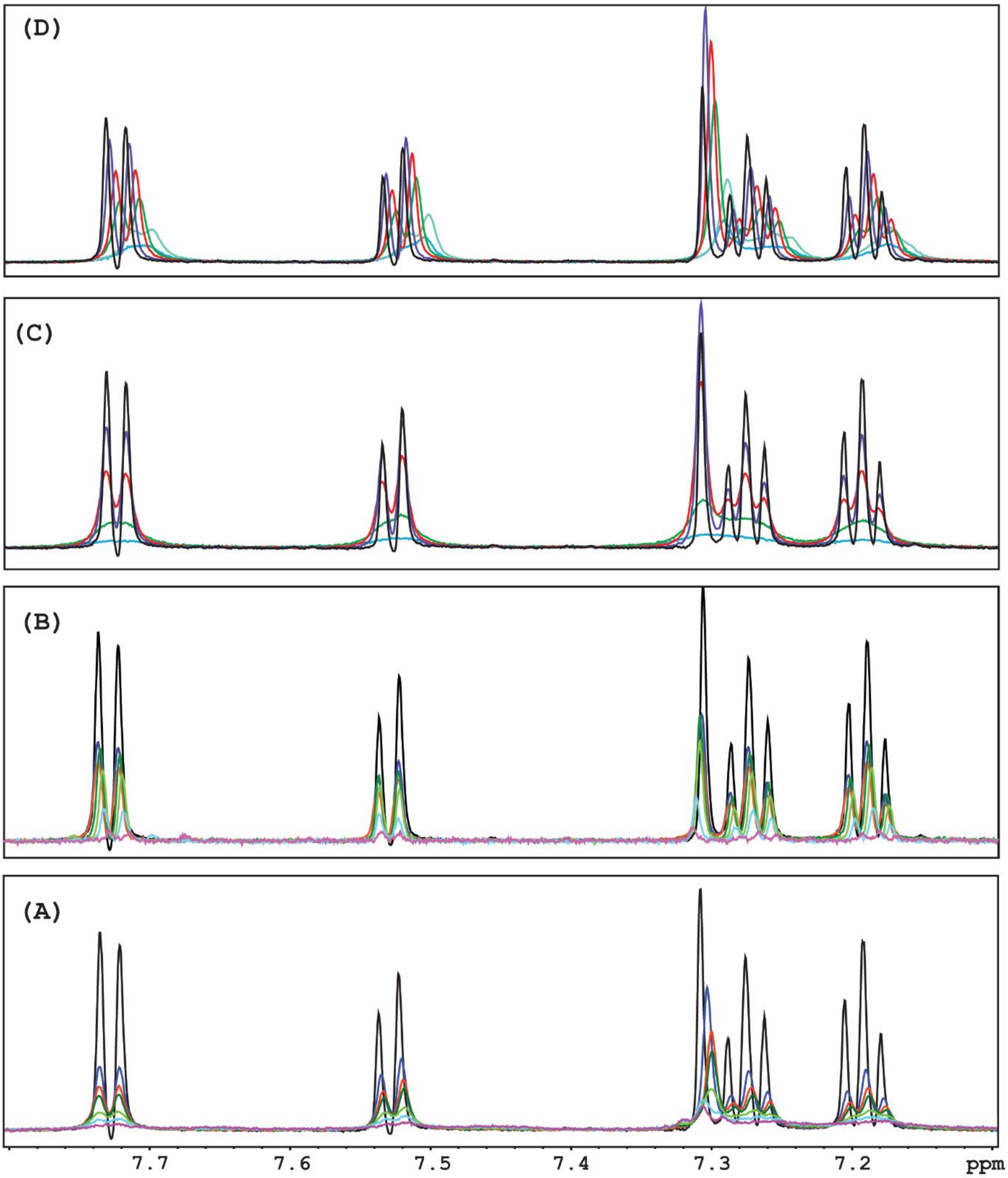


*Fig. S1. Titration of 0.5mM Trp with $TiO_2$-I(A), $TiO_2$-II (B), $CeO_2$(-)(C) and $CeO_2$(+)(D). The following colours of curves obtained at different ration of NP/Tript were used. (A), (B) Trp: $TiO_2$-I /or $TiO_2$-II: 0.5mM: 0mM (black); 0.5mM:0.031mM (blue); 0.5mM:0.0625mM (red); 0.5mM:0.125mM (green); 0.5mM:0.250mM (light green);) 0.5mM:0.5mM (light blue); 0.5mM:1.0mM (pink). (C), (D) Trp: Ce(-)(C)/or Ce(+)(D): 0.5mM: 0mM (black); 0.5mM:0.0005mM (blue); 0.5mM:0.001mM (red); 0.5mM:0.002mM (green); 0.5mM:0.005mM (light green); 0.5mM:0.007mM (light blue).*

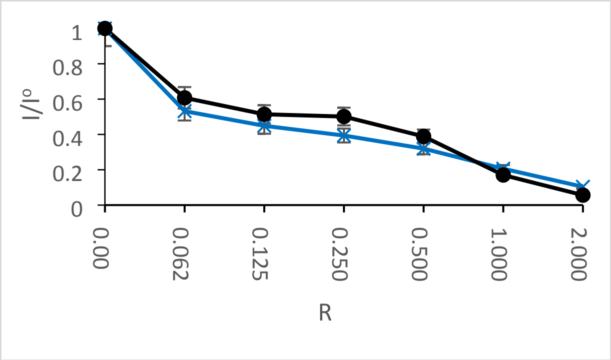


*Fig. S2. Normalized intensities (I/I°) of H6 proton resonance of Trp at 7.728 ppm vs to the ratio (R) of Trp: $TiO_2$-I (blue line) and Trp: $TiO_2$-II (black line) mixtures*

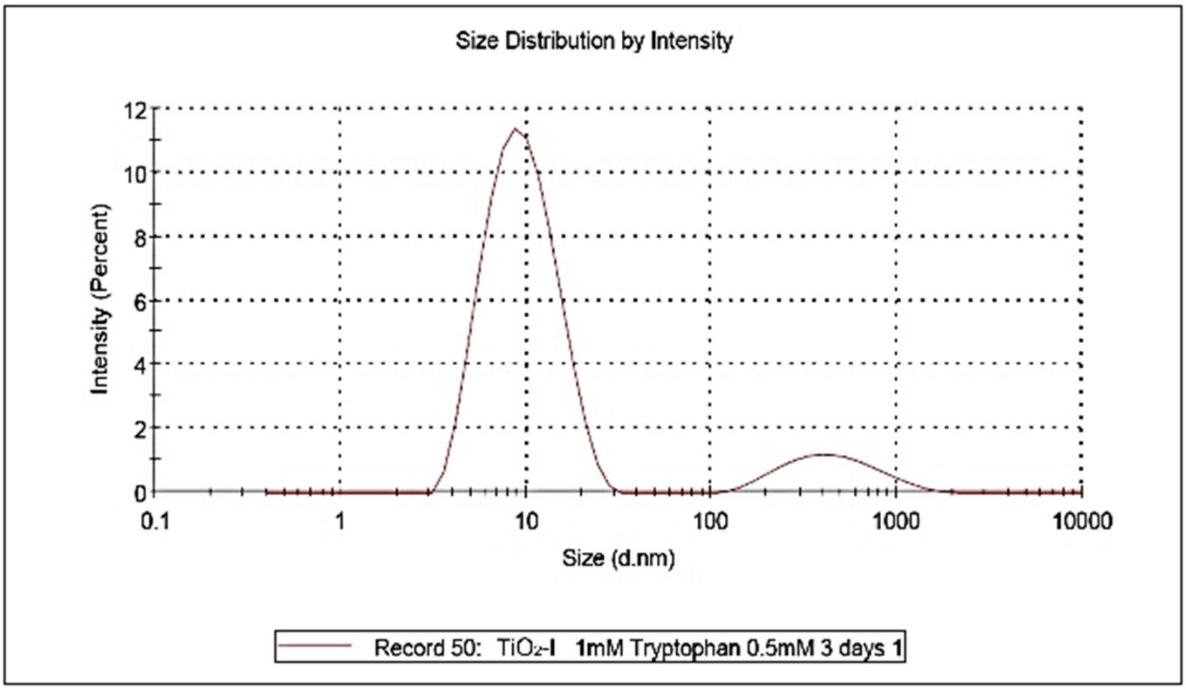


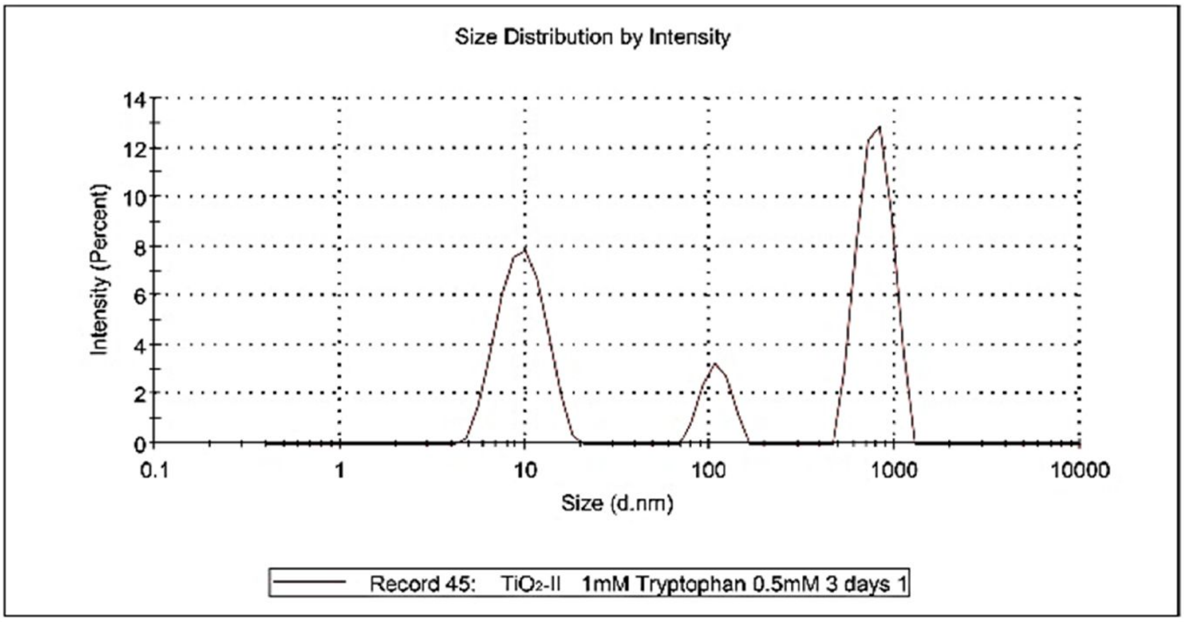


*Fig. S3. Particle size distribution by DLS after 72 h of storage for Trp solutions containing $TiO_2$-I (left) and $TiO_2$-II (right).*

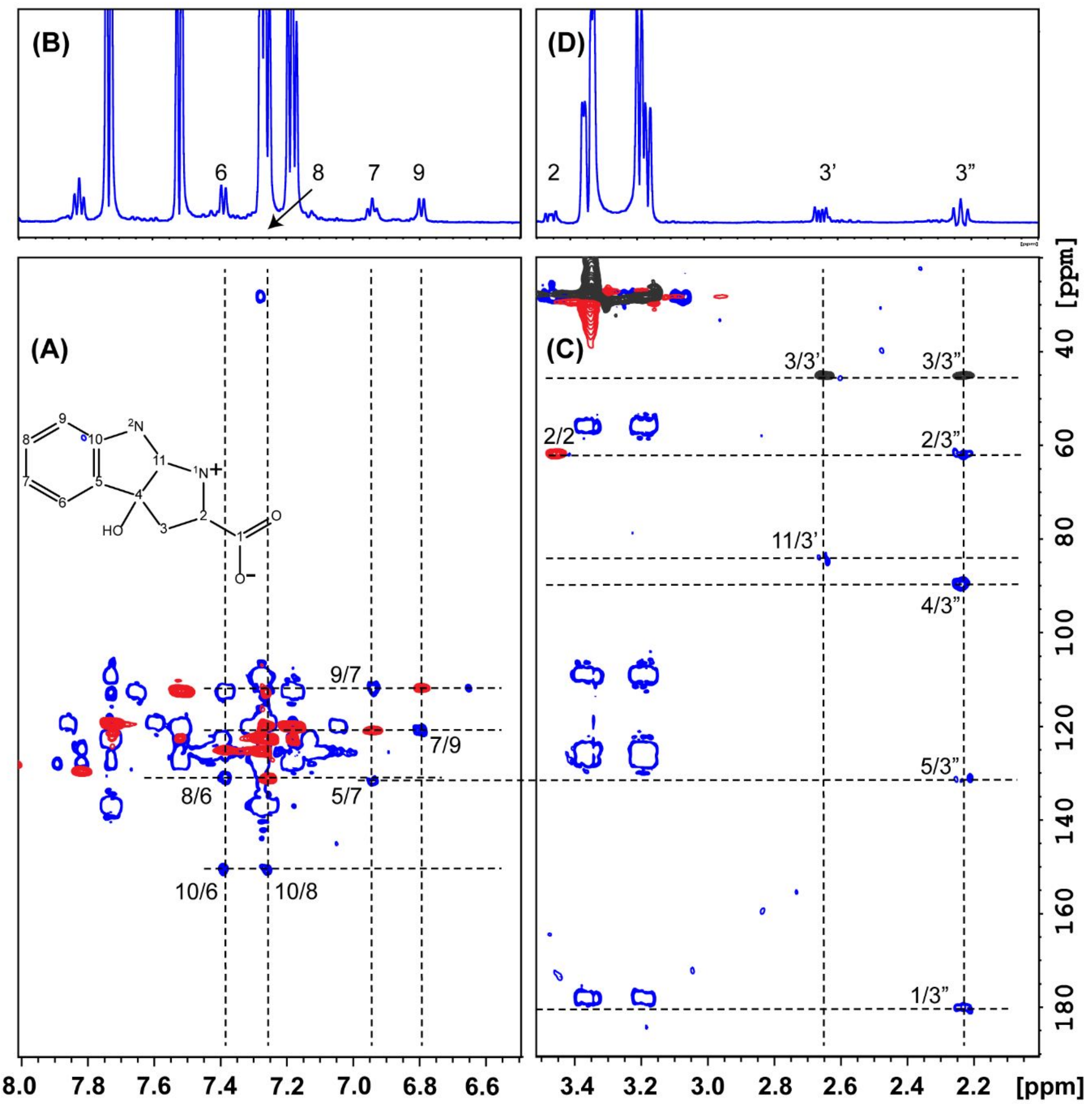


*Fig. S4.* ***NMR spectra with assignment of the oxidized product of Trp obtained in mixture with Ce(-)(C) NP****. Superposition of the HMBC (blue) and HSQC (red, black) spectra are presented in the aromatic 8.0-6.5ppm (A) and aliphatic (C) regions. 1H spectrum is in the regions (B) and (D), respectively. Assignment and numbering was shown according structure on panel (A).*

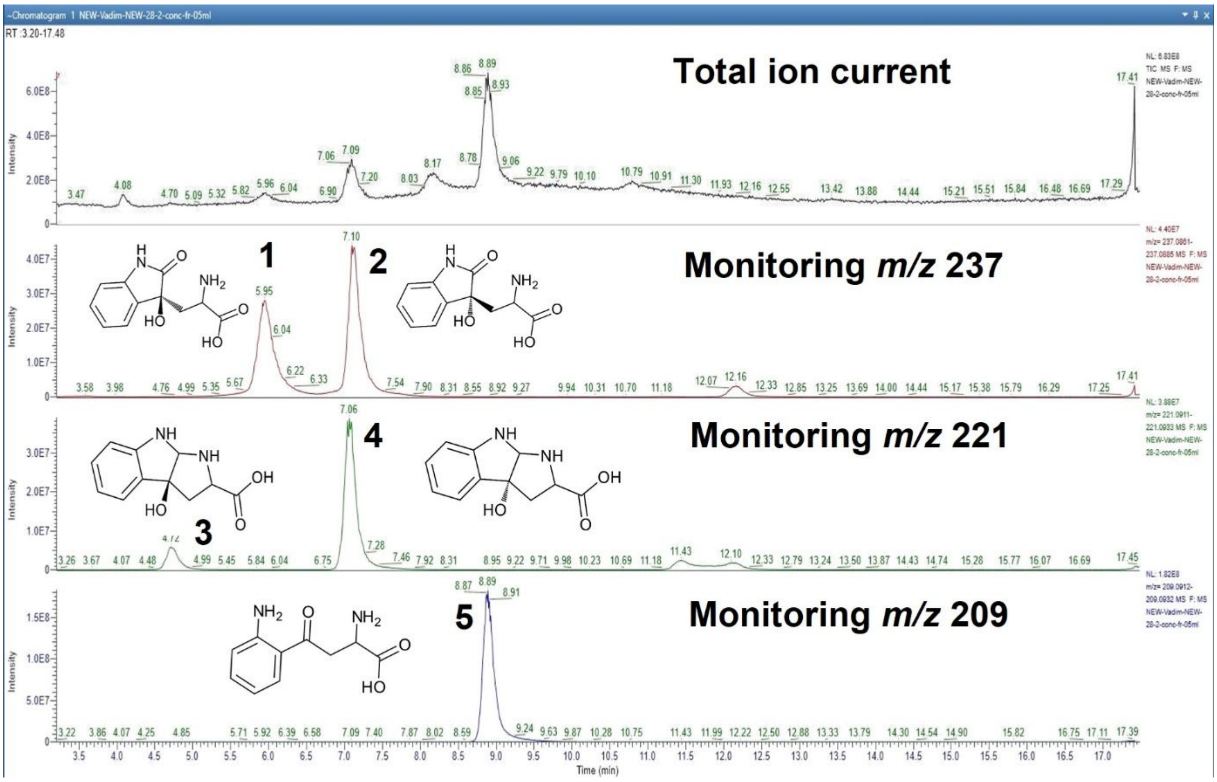


*Fig. S5. Total Ion Current (TIC) chromatogram from a Q Exactive HF mass spectrometer obtained after an HPLC-MS analysis of the sample, using a Luna Omega (4.6x100mm) C18 HPLC column, along with extracted ion chromatograms of m/z 237 (Dioxindolylalanine stereoisomers), 221 (3-Hydroxypyrroloindole carboxylic acid stereoisomers) and 209 (Kynurenine).The LC flow was diverted to waste at 17.45 min at which time unreacted Trp was eluting. Identification of ions by the HR-MS is provided below in Table TS4. Identification of the diastereoisomers was performed in relation to polarity of the molecules vs retention time in the C18 column, following methodology described in G.E. Ronsein, M.C.B. de Oliveira, M.H.G. de Medeiros, P. Di Mascio, Photochem. Photobiol. Sci. 2011, 10, 1727-1730 and references therein.*

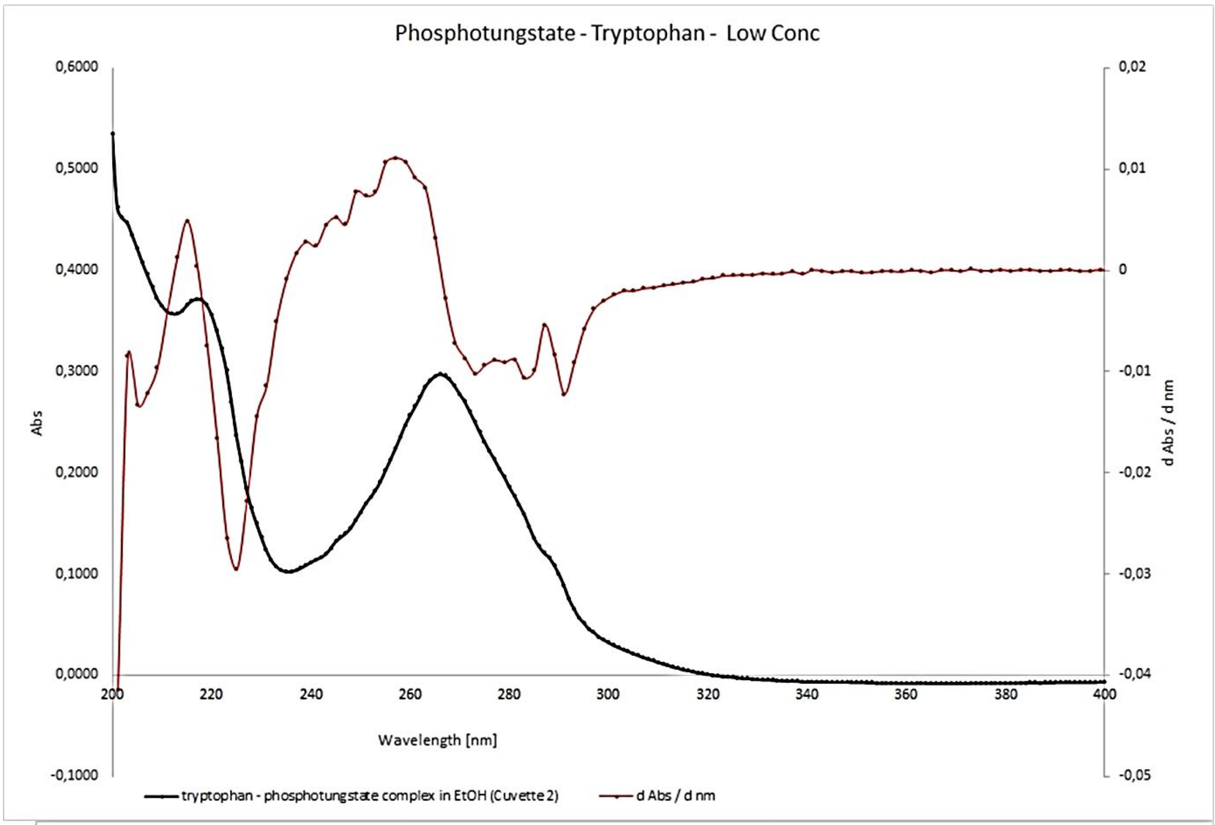


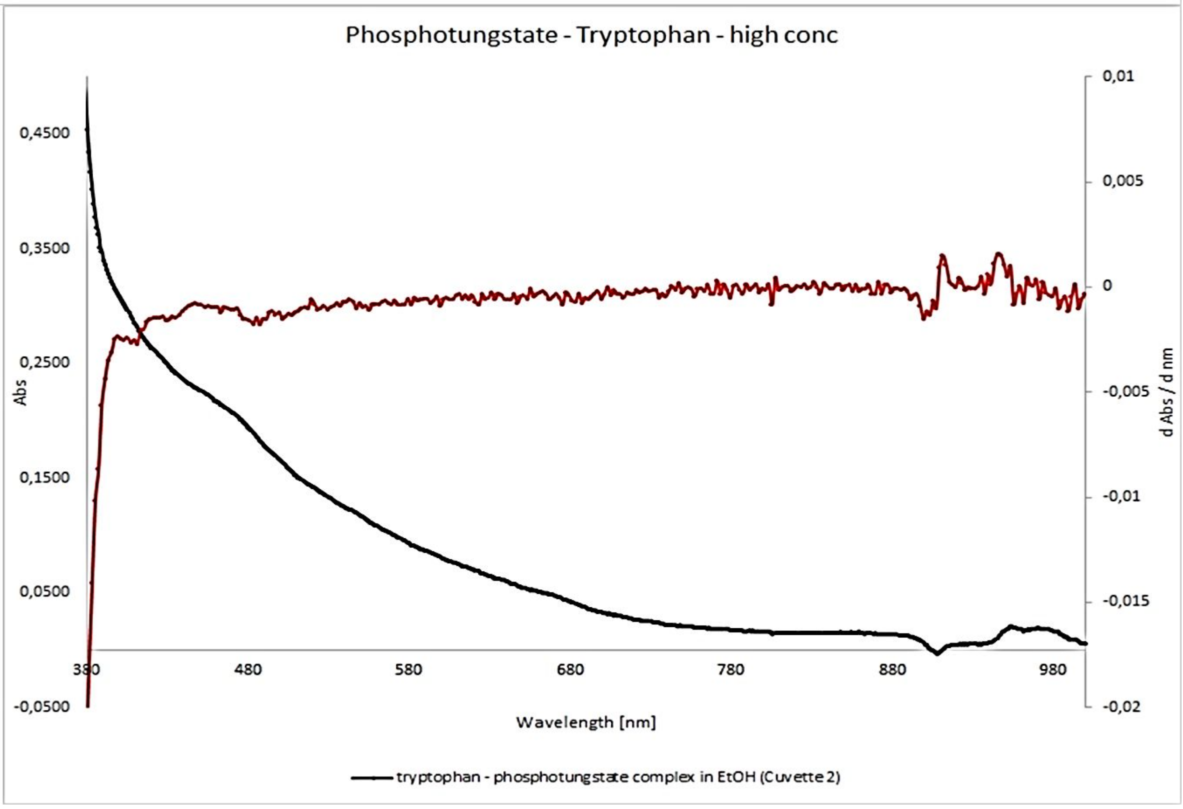


*Fig. S6. UV-Vis spectra of the EtOH:$H_2O$ = 1:1 solutions of* **$(HTrp)_3PW_{12}O_{40}\cdot5H_2O$.**

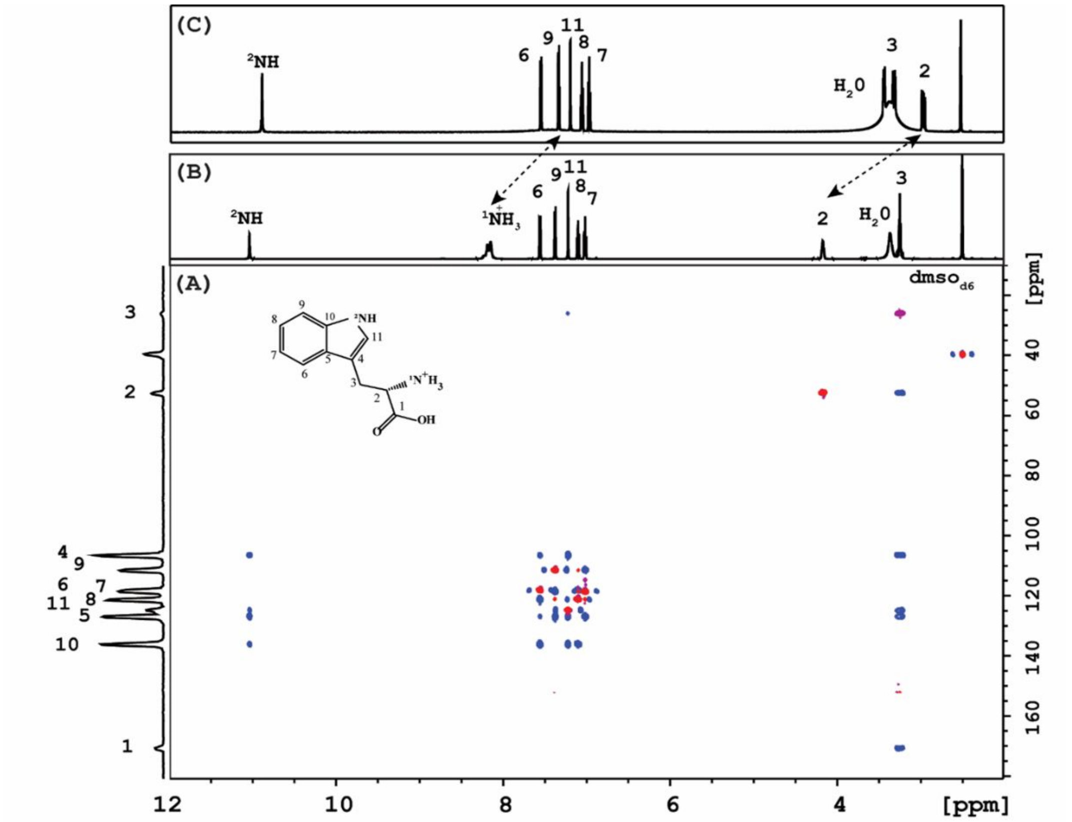


*Fig. S7 Assignment* ***of the $^1H$ and $^{13}C$ chemical shifts of Trp in complex*** **$(HTrp)_3PW_{12}O_{40}{\cdot}5H_2O$.**
*In panel (A) the superposition of the one bond $^1H$-$^{13}C$ correlation, HSQC (red), and 2, 3 $^1H$-$^{13}C$ bonds correlation, HMBC(blue), spectra dissolved in dmso$_{d6}$ are presented with $^{13}C$ projection (left) with assignment. In panel (B) and (C) 1D $^1H$ spectra of Trp in complex $(HTrp)_3PW_{12}O_{40}{\cdot}5H_2O$ and free form are presented respectively. In HMBC spectrum one bond $^1H$-$^{13}C$ coupling is not suppressed.*

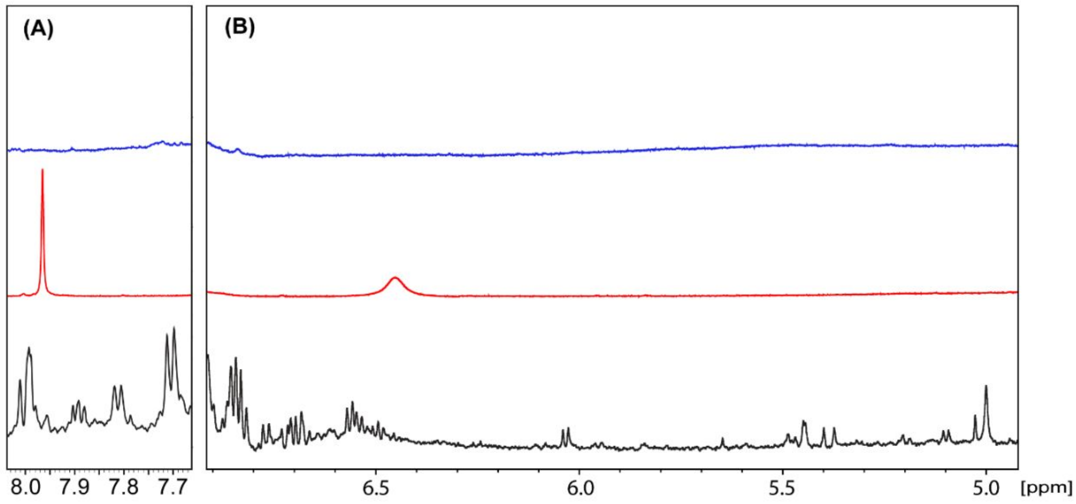


*Fig. S8* ***$^1H$ expended spectra in aromatic region (A) 8.05-7.65 (B) 6.9-4.9 ppm of the Trp:NP complex*** **exposed to UV light filtered in in dmso$_{d6}$ solution,** ***(black) Trypt: bare $TiO_2$;(blue), Trypt: TiO2-I, (red) and Trypt: TiO2-II. There are signals of oxidation products after UV irradiation in solution of Trypt: bare-$TiO_2$ but not Trypt: $TiO_2$-I or Trypt: $TiO_2$-II.***

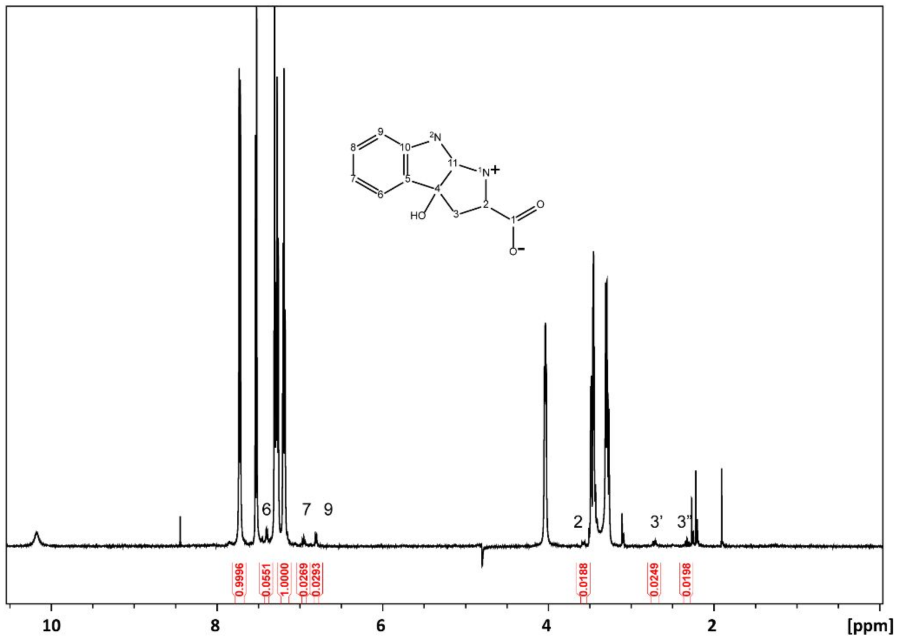


A

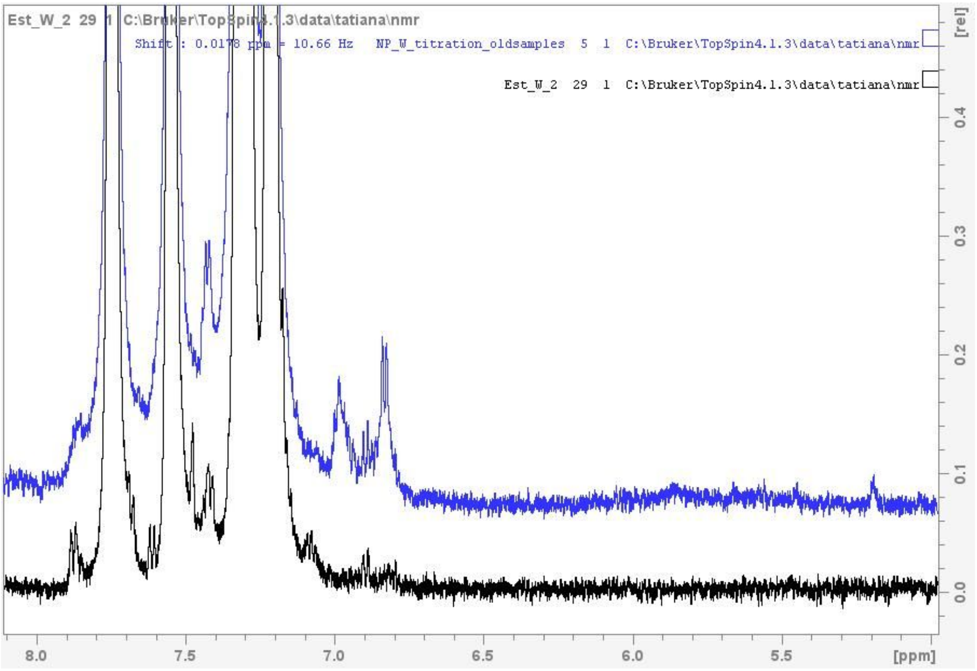


B

*Fig. S9* ***Full spectrum of the TRP, 1mg/ml, subjected to 12h oxidation in the 8 mg/ml CeO2(-) aqueous solution after centrifugation at 10000 rpm. Integration is normalized vs 1.00 for aromatic protons of residual TRP (A) and the spectrum in the aromatic proton area for a sample kept for 1 year at 5 ℃ without centrifugation, featuring formation of the same distinct product (B).***

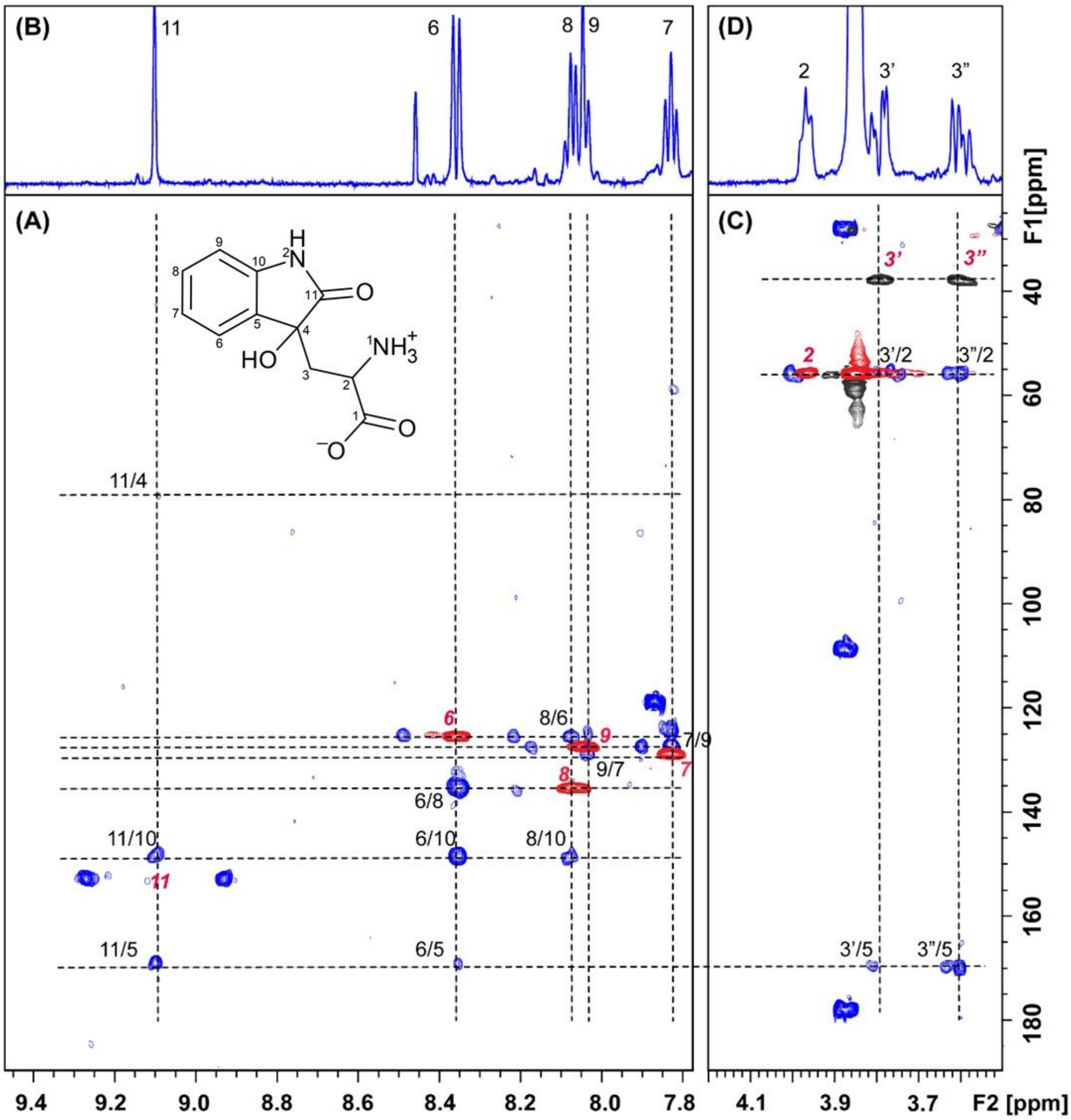


*Fig. S10* ***Identification of the second degradation product of TRP in contact with $CeO_2$(-) NP. Superposition of the HMBC (blue) and HSQC (red, black) spectra are presented in the aromatic 8.0-6.5ppm (A) and aliphatic (C) regions. $^1H$ spectrum is in the regions (B) and (D), respectively. Assignment and numbering was shown according structure on panel (A).***

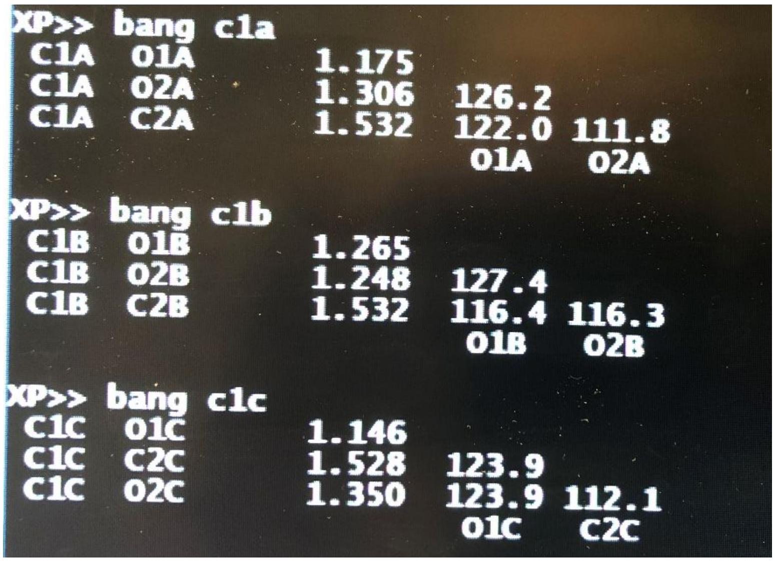


*Fig. S11* ***Bond length and angle analysis for the protonated TRP moieties in the structure of the $(HTRP)_3PW_{12}O_{40}{\cdot}5H_2O$. Of the three cations, two (a and c) are showing clearly protonated C(=O)-OH unit with asymmetric hydrogen bonding and one (b) with symmetric hydrogen bonding. Please, compare to the hydrogen bonds indicated in Fig. 5.***

# Table TS1

## Sample and crystal data for TryptPW_old1_230315.

| | | |
|---|---|---|
| **Identification code** | TryptPW_old1_230315 | |
| **Chemical formula** | $C_{33}H_{49}N_6O_{51}PW_{12}$ | |
| **Formula weight** | 3582.95 g/mol | |
| **Temperature** | 296(2) K | |
| **Wavelength** | 0.71073 Å | |
| **Crystal size** | 0.050 x 0.180 x 0.320 mm | |
| **Crystal system** | orthorhombic | |
| **Space group** | P 21 21 21 | |
| **Unit cell dimensions** | a = 14.8896(18) Å | α = 90° |
| | b = 16.565(2) Å | β = 90° |
| | c = 26.721(3) Å | γ = 90° |
| **Volume** | 6590.6(14) $Å^3$ | |
| **Z** | 4 | |
| **Density (calculated)** | 3.611 $g/cm^3$ | |
| **Absorption coefficient** | 20.987 $mm^{-1}$ | |
| **F(000)** | 6400 | |

## Table TS2

# Data collection and structure refinement for TryptPW_old1_230315.

| | |
|---|---|
| **Theta range for data collection** | 2.39 to 28.08° |
| **Index ranges** | -19<=h<=19, -21<=k<=21, -35<=l<=35 |
| **Reflections collected** | 86095 |
| **Independent reflections** | 15871 [R(int) = 0.0415] |
| **Coverage of independent reflections** | 98.9% |
| **Absorption correction** | Multi-Scan |
| **Max. and min. transmission** | 0.4200 and 0.0570 |
| **Structure solution technique** | direct methods |
| **Structure solution program** | XT, VERSION 2018/2 |
| **Refinement method** | Full-matrix least-squares on $F^2$ |
| **Refinement program** | SHELXL-2019/1 (Sheldrick, 2019) |
| **Function minimized** | $\Sigma w(F_o^2 - F_c^2)^2$ |
| **Data / restraints / parameters** | 15871 / 15 / 928 |
| **Goodness-of-fit on $F^2$** | 1.052 |
| **$\Delta/\sigma_{max}$** | 0.001 |
| **Final R indices** | 14994 data; I>2σ(I) R1 = 0.0220, wR2 = 0.0491 |
| | all data R1 = 0.0249, wR2 = 0.0501 |
| **Weighting scheme** | $w=1/[\sigma^2(F_o^2)+(0.0224P)^2+1.8325P]$ where $P=(F_o^2+2F_c^2)/3$ |
| **Absolute structure parameter** | 0.029(5) |
| **Largest diff. peak and hole** | 1.820 and -1.056 eÅ$^{-3}$ |
| **R.M.S. deviation from mean** | 0.168 eÅ$^{-3}$ |

**Table TS3. Key energies for the species involved in the reactions (1)-(3), in electron volts (eV).**

| Species | HOMO* / eV | LUMO* / eV | $E_{tot}$ / eV |
|---|---|---|---|
| $W_{12}PO_{40}^{3-}$, C1 | -3.032 | +4.215 | -113265.42 |
| $W_{12}PO_{40}^{3-}$, T | -3.032 | +4.215 | -113265.42 |
| $W_{12}PO_{40}^{3-}$(aq), C1 | -9.849 | -2.583 | -113275.81 |
| $HW_{12}PO_{40}^{2-}$, C1 | -5.648 | +1.597 | -113281.19 |
| $HW_{12}PO_{40}^{3-}$(aq), C1 | -10.103 | -2.856 | -113286.43 |
| $W_{12}PO_{40}^{4-}$ | +2.892/-0.142 | +7.135/+7.177 | -113261.58 |
| $HW_{12}PO_{40}^{3-}$ | -0.426/-2.939 | +4.335/+4.334 | -113280.31 |
| $HW_{12}PO_{40}^{3-}$(aq) | -6.589/-9.638 | -2.317/-2.295 | -113290.87 |
| TRP | -7.136 | +0.467 | -18672.74 |
| $TRP^{+}$ | -11.621/-12.111 | -4.251/-7.350 | -18665.46 |
| $TRP^{+}$(aq) | -8.484/-9.079 | -0.953/-4.100 | -18667.49 |
| $HTRP^{+}$ | -10.686 | -3.622 | -18682.81 |
| $HTRP^{+}$(aq) | -7.527 | +0.111 | -18685.22 |
| $HTRP^{2+}$ | -15.507/-15.912 | -7.907/-11.094 | -18671.89 |

*The HOMOs/LUMOs with two values correspond to doublet ground states with alpha and beta electron energies given separately.

**Table TS4 High resolution mass measurements of the molecular ions and the proposed products of oxidation of Tryptophan on the bases of their MS/MS. (data not shown).**

| Peak in EIC chromatograms | Retention time (min) | Observed [M+H]+ | Calculated [M+H]+ | Suggested elemental composition | Proposed structure |
|---|---|---|---|---|---|
| Peak#1 | 5.95 | 237.0873 | 237.0870 | $C_{11}H_{13}N_2O_4$ | 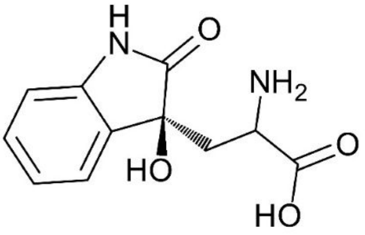 Dioxyindolealanine |
| Peak#2 | 7.10 | 237.0885 | 237.0870 | $C_{11}H_{13}N_2O_4$ | 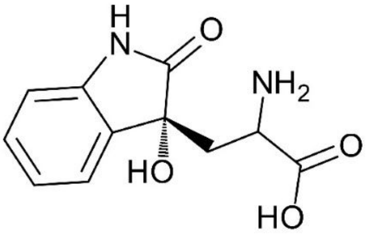 Dioxyindolealanine |
| Peak#3 | 4.72 | 221.0923 | 221.0921 | $C_{11}H_{13}N_2O_3$ | 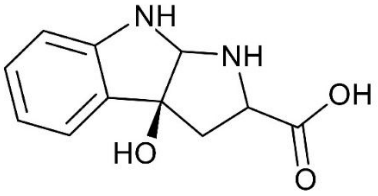 3-Hydroxypyrroloindole carboxylic acid |
| Peak#4 | 7.06 | 221.0924 | 221.0921 | $C_{11}H_{13}N_2O_3$ | 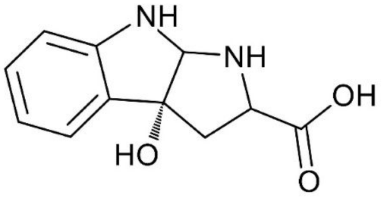 3-Hydroxypyrroloindole carboxylic acid |
| Peak#5 | 8.89 | 209.0922 | 209.0921 | $C_{10}H_{13}N_2O_3$ | 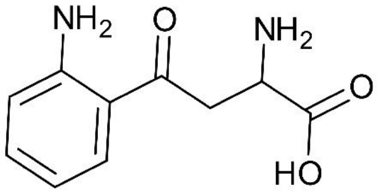 Kynurenine |
